# Surface modifications of eight-electron palladium silver superatomic alloys

**DOI:** 10.1038/s42004-022-00769-2

**Published:** 2022-11-19

**Authors:** Subrat Kumar Barik, Chih-Yuan Chen, Tzu-Hao Chiu, Yu-Rong Ni, Franck Gam, Isaac Chantrenne, Samia Kahlal, Jean-Yves Saillard, C. W. Liu

**Affiliations:** 1 Department of Chemistry, National Dong Hwa University, No. 1, Sec. 2, Da Hsueh Rd. Shoufeng, Hualien, 97401 Taiwan, ROC; 2 Department of Chemistry, Veer Surendra Sai University of Technology, Burla, Sambalpur, Odisha 768018 India; 3 Department of Chemistry, C. V. Raman Gobal University, Bidya Nagar, Bhubaneswar, Odisha 752054 India; 4 Univ Rennes, CNRS, ISCR-UMR 6226, F-35000 Rennes, France

**Keywords:** Inorganic chemistry, Synthesis and processing, Nanoparticles

## Abstract

Atomically precise thiolate-protected coinage metal nanoclusters and their alloys are far more numerous than their selenium congeners, the synthesis of which remains extremely challenging. Herein, we report the synthesis of a series of atomically defined dithiophosph(in)ate protected eight-electron superatomic palladium silver nanoalloys [PdAg_20_{S_2_PR_2_}_12_], **2a–c** (where R = O^i^Pr, **a**; O^i^Bu, **b**; Ph, **c**) *via* ligand exchange and/or co-reduction methods. The ligand exchange reaction on [PdAg_20_{S_2_P(O^n^Pr)_2_}_12_], **1**, with [NH_4_{Se_2_PR_2_}_12_] (where R = O^i^Pr, or O^n^Pr) leads to the formation of [PdAg_20_{Se_2_P(O^i^Pr)_2_}_12_] (**3**) and [PdAg_20_{Se_2_P(O^n^Pr)_2_}_12_] (**4**), respectively. Solid state structures of **2a, 2b, 3** and **4** unravel different PdAg_20_ metal frameworks from their parent cluster, originating from the different distributions of the eight-capping silver(I) atoms around a Pd@Ag_12_ centered icosahedron with *C*_2,_ *D*_3,_ *T*_*h*_ and *T*_*h*_ symmetries, respectively. Surprisingly ambient temperature crystallization of the reaction product **3** obtained by the ligand exchange reaction on **1** has resulted in the co-crystallization of two isomers in the unit cell with overall *T* (**3a**) and *C*_3_ (**3b**) symmetries, respectively. To our knowledge, this is the first ever characterized isomeric pair among the selenolate-protected NCs. Density functional theory (DFT) studies further rationalize the preferred geometrical isomerism of the PdAg_20_ core.

## Abstract (web summary)

Atomically and structurally precise silver nanoclusters hold great potential for catalytic and optoelectronic applications. Here, the authors synthesize and characterize a series of atomically defined dithio- and diselenophosphate-protected eight-electron superatomic palladium-doped silver nanoclusters, including an unprecedented pair of selenolate-protected isomers.

## Introduction

Over the last decades, the chemistry of atomically and structurally precise Au and Ag nanoclusters (NCs) and their alloys have gained a broad attention in modern science owing to their potential applications in catalysis, optoelectronics, electrochemical studies, chemical sensing, biomedicine and chiral cluster syntheses^1–9^. Size focusing synthesis in combination with atomic precision studied by X-ray crystallography sets apart these ultra-small sub-nanometer size NCs from their colloidal analogs for achieving aforementioned properties. To date, hundreds of atomically defined ligand-protected coinage metal NCs and their alloys have been synthesized. In this regard, the more common protecting ligands employed to isolate different NCs are thiols, phosphines, alkynes, hydrides or their combinations^1,10–16^. In contrast, structurally precise Au and Ag clusters co-protected by selenols are much rarer, with approximately twenty reported examples^17–22^. In order to illuminate the effects of surface functionalization of nanoparticles, recent reports have demonstrated the fabrication of several stable functional nanomaterials by using selenolates in the place of thiolates as protecting ligands. Unlike in thiolate-protected NCs, true structural isomerism, which is an interesting feature for fine tuning many properties, has not been reported so far for selenolate-protected NCs. Thus, the syntheses of selenolate-protected NCs are of paramount importance.

The initial attempt in the synthesis of a selenolate protected cluster, namely Au_25_(SeC_8_H_17_)_18_^−^, was made in 2011 by Negeshi and his coworkers *via* the reduction of an Au(I) selenolate complex by NaBH_4_^23^. Subsequently, the same group also synthesized Au_38_(SeC_12_H_25_)_24_ from Au_38_(SR)_24_ via a ligand exchange (LE) method^24^. Zhu et al. demonstrated that selenophenolate-protected Au_18_^25^ and Au_25_^11^ NCs exhibit different optical properties from those of their thiolate homologs. Later, studies on Au_24_(ER)_20_ (E = Se or S) NCs unraveled different structures and optical properties between both families of chalcogenolates^26^. In 2013, Pradeep and his co-workers reported the first atomically precise silver NC protected by selenolates, Ag_44_(SePh)_30_, which revealed similar properties as its thiolate counterpart^27^. More recently, Bootharaju *et al*. reported a Cd-doped silver NC protected by selenophenolates, namely Cd_12_Ag_32_(SePh)_36_, which exhibits rare near-infrared (NIR) photoluminescence at ∼1020 nm^18^.

As part of our research efforts in the synthesis of selenolate-protected Ag clusters, we have isolated a series of diselenophosphate (dsep) protected mono- and bimetallic silver clusters such as [Ag_7_(H){Se_2_P(O^i^Pr)_2_}_6_]^28^, [Ag_10_(Se){Se_2_P(O^i^Pr)_2_}_8_]^29^, [Ag_8_(X){Se_2_P(O^i^Pr)_2_}_6_]^+^ (X = H, Cl or Br)^30^, [Ag_11_(µ_9_-Se)(µ_3_-X)_3_{Se_2_P(O^i^Pr)_2_}_6_] (X = I^31^, Br^32^), [Ag_11_(µ_9_-I)(µ_3_-I)_3_{Se_2_P(O^i^Pr)_2_}_6_]^+ ^^33^, [Ag_12_(μ_5_-X)_2_{Se_2_P(OEt)_2_}_10_] (X = Br, I)^34^ [Ag_20_{Se_2_P(O^i^Pr)_2_}_12_]^20^, [Ag_21_{Se_2_P(OEt)_2_}_12_]^+ ^^20^, [PtAg_20_{Se_2_P(OR)_2_}_12_] (R = ^i^Pr or ^n^Pr)^21^ and [AuAg_20_{Se_2_P(OEt)_2_}_12_]^+ ^^20^. In fact the latter species, fabricated *via* a ligand exchange method, is the first structurally characterized alloy NC entirely covered by a Se ligand shell. We have produced several Pt/Pd doped Ag NCs of late^21,35,36^, among them the M_21_ core metallic system predominates (See Supplementary Table S1). Thus, we intended to outspread our approach in the development of dsep-protected PdAg_20_ alloy NCs. Amid the several synthetic methods available, the ligand exchange method^37–40^ is one of most fruitful strategies to yield molecularly pure NCs stabilized by dsep ligands. Herein we report the isolation of a series of 8-electron superatomic, dichalcogenolate-protected PdAg_20_ alloy NCs that include a pair of selenolate-protected isomers.

## Results and discussion

### Synthesis and characterization of [PdAg_20_{S_2_PR_2_}_12_], R = O^i^Pr (2a), R = O^i^Bu (2b), R = Ph (2c)

**Fig. 1:**
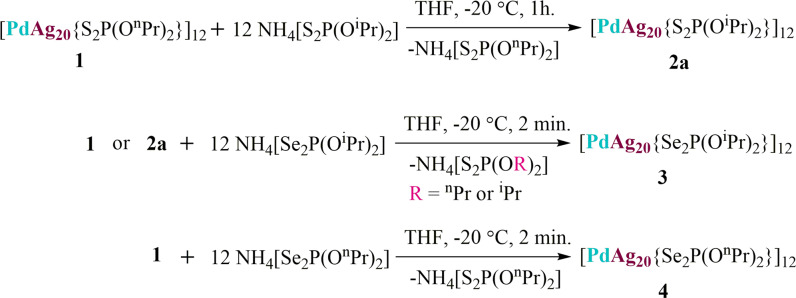
Synthesized compounds. Synthesis of dichalcogenolate protected Pd-Ag alloy NCs **2a**, **3** and **4** via ligand exchange reaction.

**Fig. 2:**
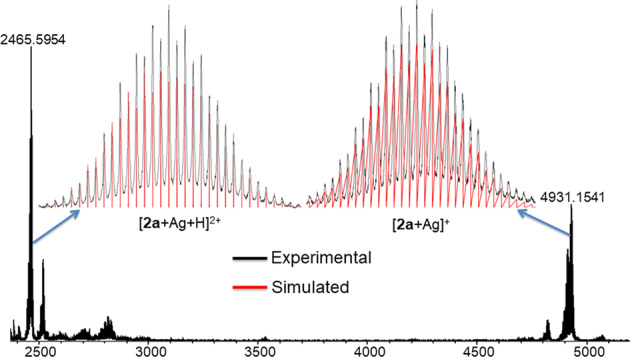
Mass spectrometry. ESI-MS (Positive mode) of [**2a** + Ag]^+^. Insets: experimental (black) and simulated (red) mass spectra.

Beforehand, we have synthesized and structurally characterized the thermodynamically stable alloy [PdAg_20_{S_2_P(O^n^Pr)_2_}_12_] (**1**). This cluster can be formally regarded as a centered icosahedral [Pd@Ag_12_]^4+^ 8-electron superatomic core (with 1S^2^ 1P^6^ 1D^0^ superatomic configuration) passivated by an outer shell made of eight Ag^+^ and twelve monoanionic dithiophosphate (dtp) ions in such a way the complete PdAg_20_ metallic kernel is lowered to ideal *C*_2_ symmetry and the whole NC to *C*_1_^36^. NC **1** is robust, yet the liability of its protecting ligands tempted us to study its ligand exchange (LE) behavior. As shown in the Fig. 1, the treatment of **1** with 12 equivalents of NH_4_[S_2_P(O^i^Pr)_2_] at −20 °C in tetrahydrofuran (THF) led to the formation of [PdAg_20_{S_2_P(O^i^Pr)_2_}_12_] (**2a**) in 70% yield within an hour. There was no obvious color change observed when NH_4_[S_2_P(O^i^Pr)_2_] was added to the brown red solution of **1** in THF during the course of reaction, thus the progress of reaction was monitored by thin layer chromatography (TLC). Alternatively, the same compound was produced more conveniently by direct archetypal one pot synthetic method in moderate yield (41 %) (See Experimental methods). In parallel to the synthesis of **2a**, compound [PdAg_20_{S_2_P(O^i^Bu)_2_}_12_] (**2b)** was synthesized via co-reduction method in 40% yield. Compound [PdAg_20_{S_2_P(OPh)_2_}_12_] (**2c**) was synthesized via the LE method in 65% yield (Experimental methods). All NCs (**2a–c**) have been characterized by positive ion mode electrospray ionization mass spectrometry (ESI-MS) and nuclear magnetic resonance (NMR) spectroscopy. ESI mass spectra of **2a–c** have been provided in Table 1, Fig. 2 and Supplementary Figs. S1 and S2. ^31^P{^1^H} and ^1^H NMR spectra of **2a–c** have been provided in Table 1 and Supplementary Data 1, Figs. 1–5.

**Table 1:** Spectroscopic data of compounds for **1**, **2**(**a–c**), **3** and **4**.

Compound	^31^P NMR(ppm)	UV–vis(nm)	Emission (nm)^a^	ESI-MS(*m*/*z*, M^+^)
**1**	104.9	384, 436	748	4930.8
**2a**	101.66	384, 436	741	4931.15
**2b**	103.85	384, 436	732	5267.64
**2c**	63.11	419, 486	669	2735.76^b^
**3**	68.16	410, 501	712	6055.77
**4**	73.59	408, 498	702	6056.02

^a^Photoluminescence recorded in 2-Methyl tetrahydrofuran at 77 K.

^b^ESI-MS peak corresponds to [M + 2Ag]^2+^ peak.

**Fig. 3:**
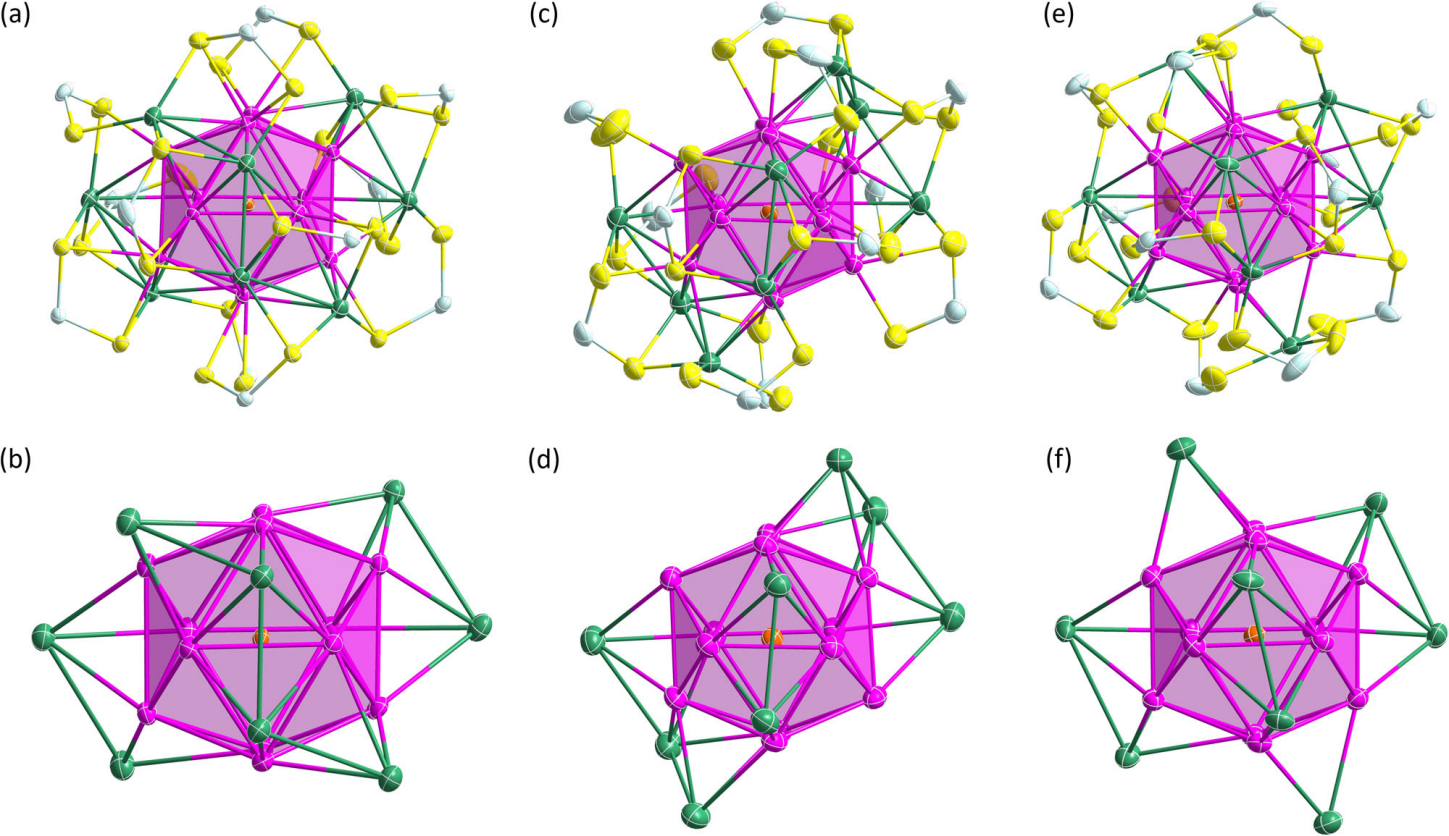
Molecular structures of 1, 2a and 2b. **a**, **b** Total structure of **1** (propoxy groups omitted for clarity) and its Pd@Ag_20_ metallic core with *C*_2_ symmetry, respectively. **c**, **d** Total structure of **2a** (isopropoxy groups omitted for clarity) and its Pd@Ag_20_ metallic core with *C*_2_ symmetry, respectively^36^. **e**, **f** Total structure of **2c** (isobutoxy groups omitted for clarity) and its Pd@Ag_20_ metallic core with *D*_3_ symmetry, respectively. (color code. Pd: orange; Ag_ico_: pink, Ag_cap_: green; S: yellow; P: sky blue).

**Fig. 4:**
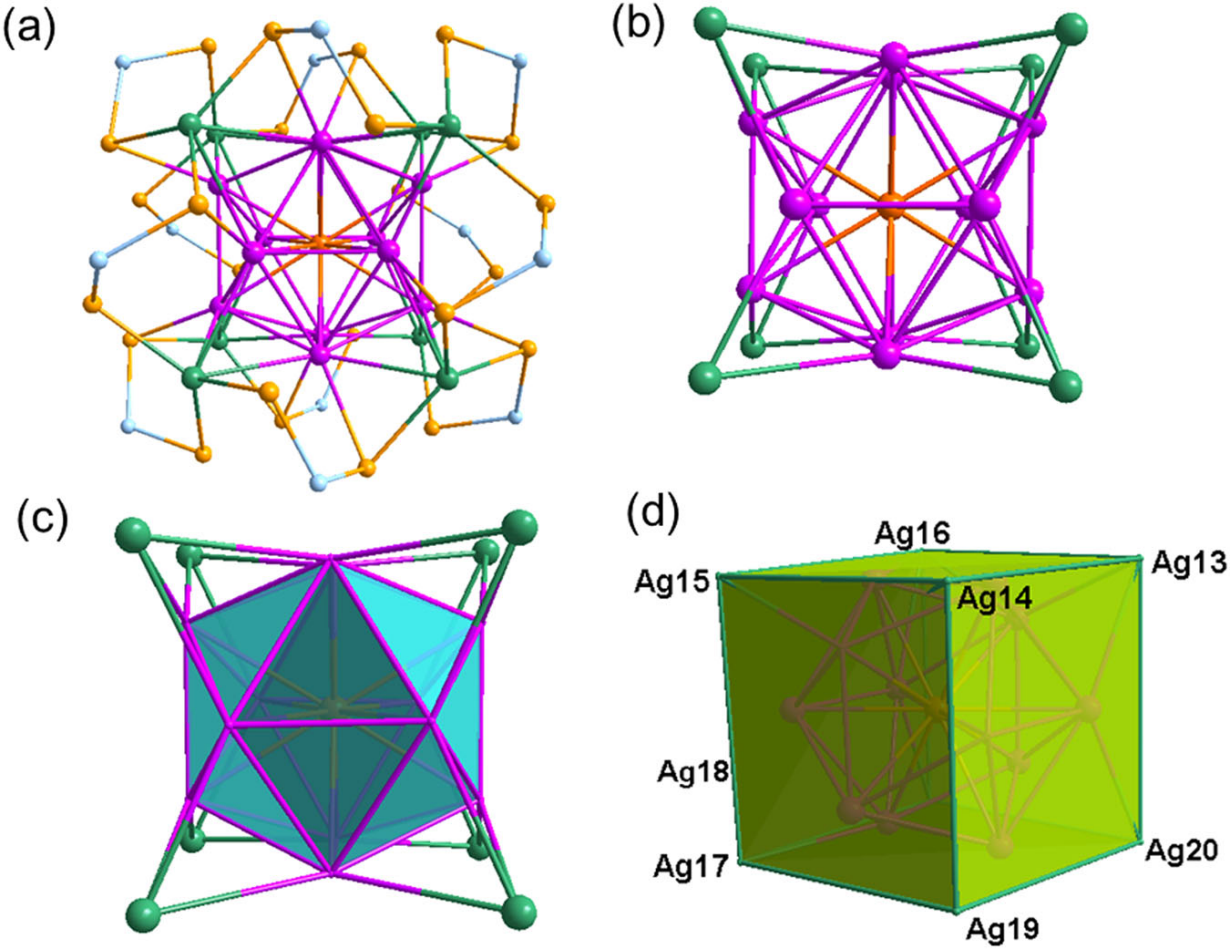
Molecular structure of 3(*Pn*). **a** Total structure of [PdAg_20_{Se_2_P(O^i^Pr)_2_}_12_] (**3**(*Pn*)) (isopropoxy groups omitted for clarity), **b** Illustration of the Pd@Ag_20_ metallic core in **3**(*Pn*) with *T*_h_ symmetry, **c** A view of the Pd@Ag_12_ centered icosahedron core with its 8 capping Ag atoms, **d** The centered Pd@Ag_12_ icosahedron inscribed in Ag_8_ cube (color code. Pd: orange red; Ag_ico_: pink, Ag_cap_: green; Se: light orange; P: light blue).

**Fig. 5:**
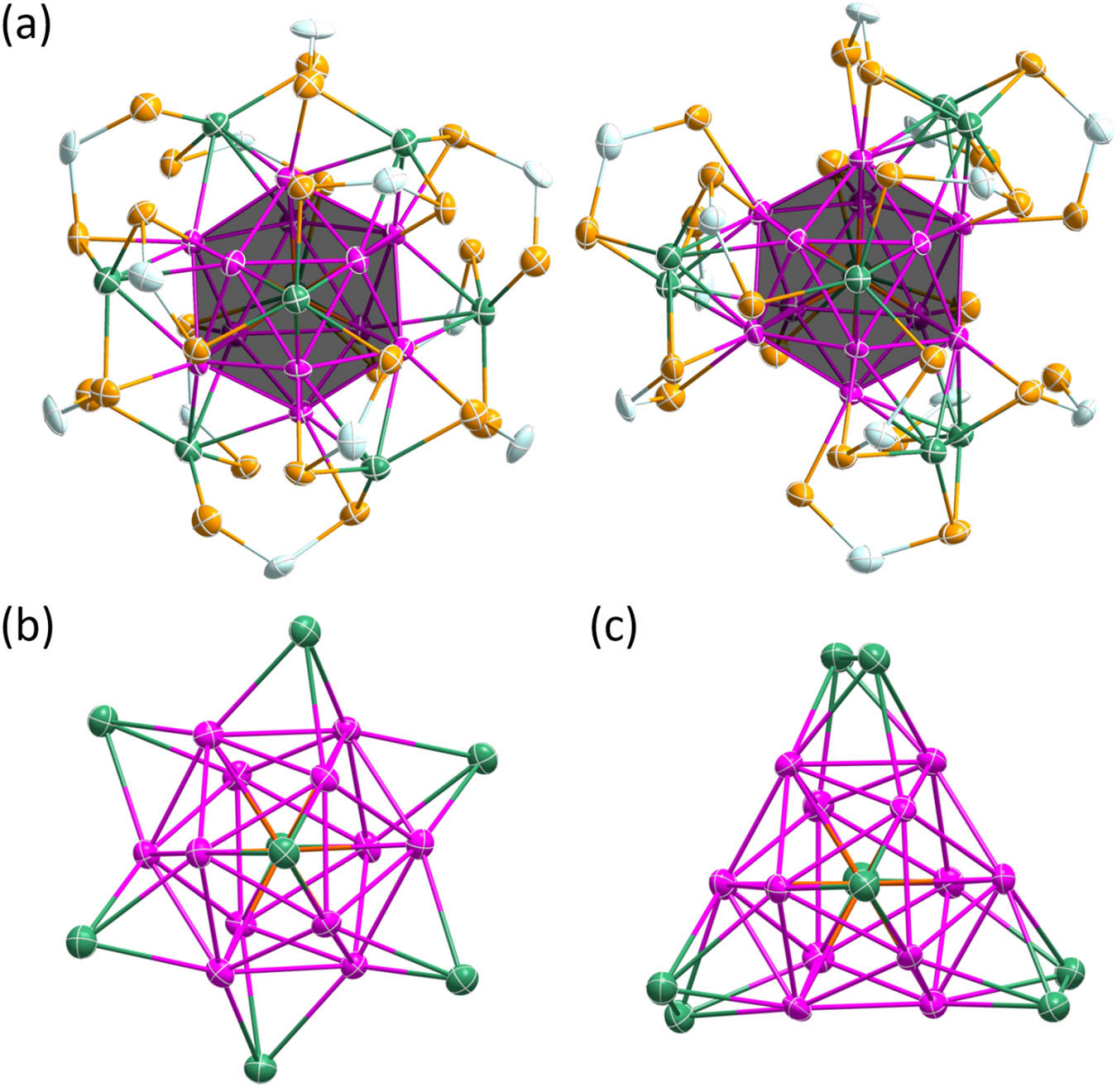
The molecular structure of 3a and 3b. **a** The two co-crystallized structures of [PdAg_20_{Se_2_P(O^i^Pr)_2_}_12_], **3a** (left) and **3b** (right). Isopropoxy groups have been omitted for better clarity. **b** The *T*_*h*_ Pd@Ag_20_ metallic core of **3a**. **c** The *C*_3_ Pd@Ag_20_ metallic core of **3b**. (color code. Pd: orange; Ag_ico_: blue, Ag_cap_: pink; Se: light orange; P: dark green).

Upon the replacement in **1** of the dithiophosphates ligands having linear alkyl chain (n-propyl) with their branched derivatives (di-isopropyl dithiophosphates), the ^31^P{^1^H} NMR spectrum in CDCl_3_ displays a signal shift from 104.9 ppm to 101.66 ppm at room temperature (Supplementary Fig. S3). The ^1^H NMR spectrum of **2a** in CDCl_3_ shows two set of signals with a multiplet ranged at δ = 4.97–4.85 ppm (corresponding to the –OC*H* groups) and a doublet ranged at δ = 1.35–1.33 ppm (corresponding to –(C*H*_3_)_2_), in an integration ratio of 1:6 which is clearly attributed to the ^i^Pr groups (Supplementary Data 1, Fig. S2).

The ESI mass spectrum (positive-ion mode) was recorded to identify the molecular formula of **2a**. The spectrum reveals two prominent bands corresponding to [**2a** + Ag]^+^ at *m*/*z* 4931.15 (calcd. 4930.97), and [**2a** + Ag+H]^2+^ at *m*/*z* 2465.59 (calcd. 2465.95). Their simulated isotopic distributions are in good agreement with the experimental results (Fig. 2). The position of the molecular ion peak in **2a** matches exactly with its parent NC **1**, signifying the retention metal atomicity upon LE. Moreover, the UV–vis absorption spectrum of **2a** features the same absorption pattern (384, 436 nm) as its parent NC **1**. Thus, from the above spectroscopic data obtained in solution state, one would presume that the structure of **2a** is the same as **1**.

Similarly, the ^31^P{^1^H} NMR spectrum displays one type of resonance for **2b** (δ = 103.8 ppm) and for **2c** (δ = 63.1 ppm). The ^1^H NMR spectra of **2b** and **2c** displayed three and two types of resonances, corresponding to iso-butoxy and phenyl groups, respectively. Further, the positive ion mode ESI mass spectra of **2b** and **2c** show prominent bands corresponding to [M + Ag]^+^ at *m/z* 5267.64 (Calcd. 5267.62) and [M + 2Ag]^2+^ at *m/z* 2735.7615 (Calcd. 2735.8916), respectively. In order to elucidate the structure of these nanoalloys (**2a**–**c**), single crystals X-ray diffraction studies were undertaken. We were successful to crystallize **2a** and **2b**. The details of their X-ray structural analysis were discussed below. All of our attempts to crystallize **2c** were failed.

Single crystals of suitable quality for X-ray diffraction for **2a** and **2b** were grown by crystallization from diffusion of hexane into a concentrated dichloromethane solution at −4 °C within couple of weeks. Surprisingly, the resulting solid-state structures unveil different configuration of the outer shell which protects the 8-electron [Pd@Ag_12_]^4+^ core, as illustrated in Fig. 3 compared to that of **1**. In particular, the arrangements of the 8 Ag^+^ capping atoms around the centered icosahedral core differs from that in **1**, as one can see in Fig. 3 (from Fig. 3b→3d→3f). Whereas in both **1** and **2a** NCs the PdAg_20_ skeleton adopts *pseudo-C*_2_ symmetry; that of **2b** the PdAg_20_ skeleton adopts *pseudo*-*D*_3_ symmetry (Supplementary Fig. S4). The twelve dtp ligands in **2a** are equally distributed on both sides of the *pesudo*-*C*_2_ axis (Supplementary Fig. S4). These dtp ligands are coordinated to both icosahedral and capping silver atoms (Ag_ico_ and Ag_cap_, respectively) in five different binding modes bimetallic biconnectivity (η^2^: µ_1_, µ_1_), bimetallic triconnectivity (η^2^: µ_2_, µ_1_), trimetallic triconnectivity (η^3^: µ_2_, µ_1_), trimetallic tetraconnectivity (η^3^: µ_2_, µ_2_) and tetrametallic tetraconnectivity (η^4^: µ_2_, µ_2_) (Supplementary Fig. S5) in a ratio of 1:1:7:1:2. Further the seven dtp ligands with trimetallic triconnectivity (η^3^: µ_2_, µ_1_), differ in the coordination to different combination of Ag_cap_ and Ag_ico_ atoms except for a couple of dtp ligands (red box, in Supplementary Fig. S5). As in any 8-electron dtp- or dsep-protected M_21_ NC characterized so far, the eight Ag^+^ capping atoms in **2a** lie in a nearly planar AgSe_3_ coordination mode, making locally stable 16-electron metal centers. With the 12 protecting ligands around the PdAg_20_ metal skeleton (Fig. 3c), the entire molecular symmetry of **2a** is *C*_1_. On the other hand, the total twelve dtp ligands in **2b** are distributed in three spherical rows around the *pseudo*-*C*_3_ axis in 3:6:3 ratios (Supplementary Fig. S4). They bind to both capping and icosahedral silver atoms only through two coordination patterns that are trimetallic triconnectivity and trimetallic tetraconnectivity (Supplementary Fig. S6), in such a way the whole NC ideal symmetry is reduced to *C*_3_. A similar *C*_3_ arrangement has been described in the related 8-electron NC [Ag_21_{S_2_P(O^i^Pr)_2_}_12_]^+ ^^41^.

The inner icosahedral Pd@Ag_12_ cores of **2a** and **2b** are very similar to that of **1**. The Pd-Ag radial bond distances average 2.755 Å, 2.767 Å for **2a** and **2b**, respectively (2.757 Å in **1**) and the peripheral Ag_ico_-Ag_ico_ and Ag_ico_-Ag_cap_ bond distances in **2a** and **2b** are also fairly similar to those of **1** (Table 2). Thus, the 8-electron nanoalloys **1, 2a** and **2b** whose compositions differ only by the nature of their alkyl substituents, can be considered as *pseudo*-isomers. The presence of different arrangements of their outer shells is likely the result of the slightly different steric factors of their alkyl chains in **1** and **2a**.

**Table 2:** Selected structural metrics (average (top line) and ranges (bottom line)) of compounds for **1**, **2a-b**, **3, 3a-b** and **4**.

Entry		Ag_ico_-Ag_ico_	Ag_ico_-Ag_cap_	Ag_ico_-E	Ag_cap_-E	E-E
**1**		2.897	2.971	2.687	2.540	3.414
		2.827-2.987	2.856-3.346	2.471-3.047	2.480-2.726	2.772-2.747
**2a**		2.896(7)	3.020(7)	2.605(14)	2.542(19)	3.399
		2.807(1)− 2.997(1)	2.863(1)−3.287(1)	2.486(4)−2.933(4)	2.472(5)−2.651(4)	3.304-3.456
**2b**		2.893(6)	3.014(5)	2.542(2)	2.506(1)	3.400 (3)
		2.829(8)− 2.930(1)	2.847(9)−3.204(7)	2.480(3)−2.590(2)	2.330(1)−2.590(3)	3.380(4)−3.430(2)
**3** (*Pn*)		2.902(11)	2.949(100)	2.683(10)	2.614(15)	3.688
		2.845(2)−2.969(2)	2.899(2)−3.005(2)	2.666(3)−2.696(3)	2.595(3)−2.639(3)	3.655-3.704
**3**(*P*3_1_c)	**3a**	2.901(9)	2.950(8)	2.668(7)	2.607(10)	3.665
		2.841(2)−2.957(3)	2.901(3)−2.989(2)	2.662(4)−2.674(3)	2.600(3)−2.617(3)	3.646-3.691
	**3b**	2.896(10)	2.965(9)	2.739(8)	2.607(12)	3.668
		2.845(3)−2.945(2)	2.880(3)−3.123(3)	2.628(3)−3.104(4)	2.576(3)−2.673(3)	3.653-3.694
**4**		2.893(2)	2.949(2)	2.668(2)	2.618(2)	3.698
		2.833(2)−2.961(2)	2.902(2)−3.009(2)	2.630(2)−2.699(2)	2.607(2)−2.637(2)	3.677-3.721

#### [PdAg_20_{Se_2_P(O^i^Pr)_2_}_12_], (3) and its 3(*Pn*) solid-state structure

After successful isolation of the **2a–c** NCs, it was indeed obvious to attempt the synthesis of their diselenophosphate (dsep) protected analogs *via* LE reactions. Compound **1** was treated with NH_4_[Se_2_P(O^i^Pr)_2_] at −20 °C in THF (Fig. 1). The reaction proceeded immediately as the color of the reaction mixture altered from brown red to purple which indicates replacement of surface dtp ligands by dsep ligands (Supplementary Fig. S7). The possibility of partial replacement of ligands cannot be excluded, however we have never encountered the partial replacement when we intend to produce dsep protected NCs from their dtp siblings via LE method^20–22,28–34^. In particular, most of the cases these reactions are associated with alteration of the metallic cores^20–22,28–34^. The change in metallic core is certainly a reason for the distinct color change, however we believe the change in the ligand environment plays a major role for the immediate color change. Note that, when we performed ligand exchange reaction onto **1** with n-propyl dithiophosphate surface ligand by iso-propyl dithiophosphate ligand, then there was no remarkable color changes observed, even though reaction culminated in the formation of altered metal core. The positive ion ESI mass spectrum of reaction mixture shows a prominent band or molecular ion peak at *m/z* = 6055.77 (calcd. 6055.23) corresponding to [PdAg_20_{Se_2_P(O^i^Pr)_2_}_12_ + Ag]^+^ (Supplementary Fig. S8).

In order to determine its molecular structure, much effort was devoted to obtain suitable crystals for single crystal X-ray diffraction. Several sets of crystallization with varied solvent combinations and in mutable temperatures ended up producing extremely bad quality crystals. Nevertheless, diffusion of hexane into a saturated acetone solution of the reaction mixture kept at −10 °C yielded proper single crystals within a week. The crystals of **3** obtained from this low temperature crystallization were dissolved in *d*_6_-acetone and subjected to ^31^P{^1^H} and ^1^HH NMR studies. The ^31^P{^1^H} NMR spectrum at ambient temperature shows a single resonance at δ = 68.16 ppm (161.9 MHz, *d*_6_-acetone) flanked with two set of satellites (^1^*J*_P-Se_ = 604.51 and 710.40 Hz) (Supplementary Data 1, Fig. 6). ^1^H NMR spectrum shows characteristic signals of isopropyl ligands of di-isopropyl diselenophosphates (Supplementary Data 1, Fig. S7).

**Fig. 6:**
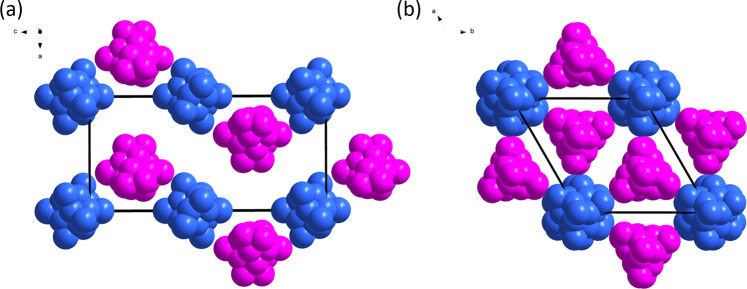
Illustration of packing diagrams of the co-crystallized NCs 3a and 3b with dsep ligands omitted for clarity. **a** View down the *b* axis. **b** View down the *c* axis (color code: *T* layers are in blue and *C*_3_ layers in pink.).

**Fig. 7:**
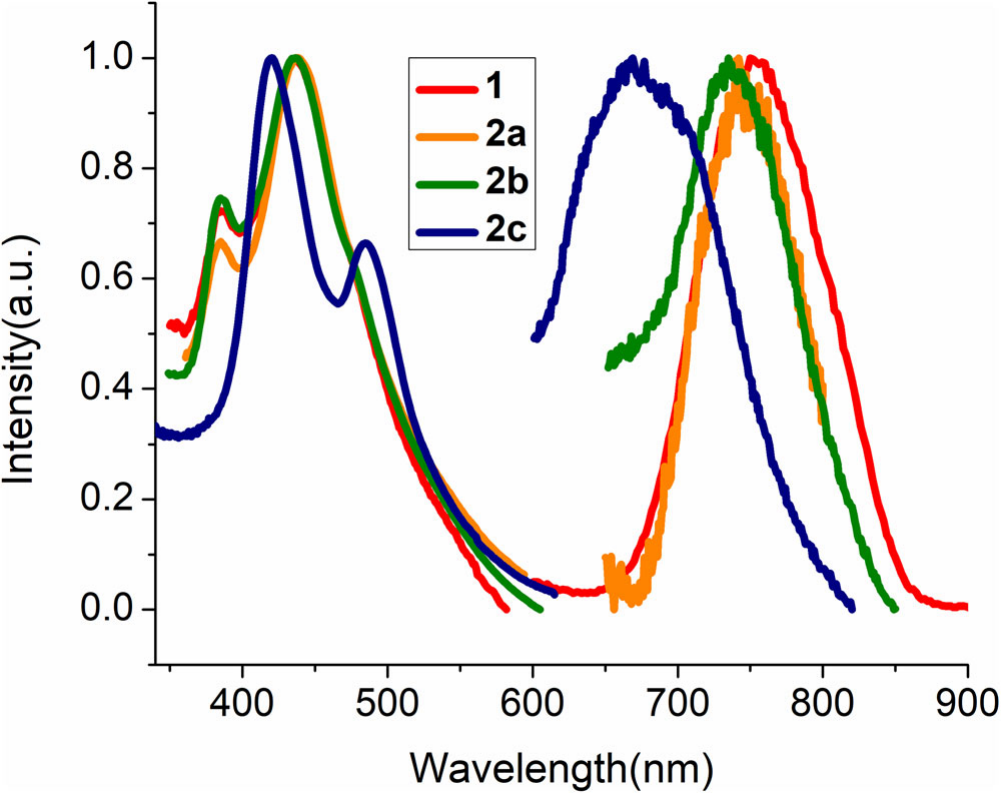
Photophysical properties. UV–vis spectrum (left) of **1**, **2a**, **2b** and **2c** in 1 ×10^−5 ^M CH_2_Cl_2_ and the normalized emission spectrum (right) of **1**, **2a**, **2b** and **2c** in MeTHF at 77 K.

Single crystals obtained were subjected to the X-ray diffraction study. Their analysis reveals that **3** crystallize in *Pn* space group. Its solid-state structure is labeled **3**(*Pn*) in the following. It is shown in Fig. 4 and exhibits a Pd-centered icosahedral Ag_12_ core inscribed in a cube made up of 8 capping Ag atoms, in such a manner that the entire Pd@Ag_12_@Ag_8_ framework attains ideal *T*_*h*_ symmetry. The whole metal kernel is protected by 12 dsep ligands situated on the 12 edges of the cube, in such a way that the whole NC ideal symmetry is reduced to *T*. The detailed molecular structure of **3**(*Pn*) is identical to that of **3a**, discussed in the next section below.

It should be also mentioned that the molecularly pure as-synthesized NC **2a** also was used as starting precursor for LE reaction in order to produce **3**. Therefore, the reaction of **2a** with NH_4_[Se_2_P(O^i^Pr)_2_] at −20 °C in THF was performed (Fig. 1). Likewise, in the transformation from **1** to **3** the solution undergoes an instant color change from brown to purple. The work up of the reaction mixture was done immediately. The ^31^P{^1^H} NMR spectrum of the reaction mixture exclusively shows single resonance at δ = 68.16 ppm in *d*_6_-acetone, the same resonance as observed in ^31^P{^1^H} NMR spectrum of **3**. Thus, the transformation from **2a** to **3** was confirmed by ^31^P{^1^H} NMR spectroscopy.

#### The solid-state structure of [PdAg_20_{Se_2_P(O^i^Pr)_2_}_12_], 3(*P*3_1_*c*)

Single-crystals of **3** could also be obtained by slow diffusion in hexane into the saturated acetone solution of reaction mixture at ambient temperature. Their X-ray analysis reveals that in such conditions **3** crystallizes in the *P*3_1_*c* space group. This solid-state phase is labeled **3**(*P*3_1_*c*) in the following. It reveals an isomeric NC pair of [PdAg_20_{Se_2_P(O^i^Pr)_2_}_12_] clusters (**3a** and **3b**), co-crystallized in the unit cell in a 1:1 ratio, with *T* and *C*_3_ *pseudo*-symmetry, respectively (Fig. 5)^42^. The molecular structure of **3a** (*T* symmetry) is the same as that of **3**(*Pn*), as well as that of the previously reported isoelectronic monocationic [MAg_20_{Se_2_P(R)_2_}_12_]^q+^ (M = Ag or Au; R = OEt; q = 1: M = Pt; R = O^i/n^Pr; q = 0)^20,21^. The structural metrics of **3a** and **3**(*Pn*), are similar (Table 2). Their icosahedral Pd@Ag_20_ core embedded within a cuboid made of eight capping Ag atoms, resulting in a PdAg_20_ framework of *T*_h_ symmetry, is shown in Fig. 4b–d. Their twelve dsep ligands display trimetallic triconnectivity (η^3^: µ_2_, µ_1_) bridging pattern with two capping Ag atoms (Ag_cap_) and one icosahedral Ag atom (Ag_ico_), reducing the whole ideal NC symmetry to *T*. The molecular structure of **3b** (*C*_3_ symmetry) is similar to that of **2b** (see above) and [Ag_21_{S_2_P(O^i^Pr)_2_}_12_]^+ ^^41^. The differences in the positions of the outer capping Ag atoms in **3a** and **3b**, and their possible interchange pathway, is illustrated in Supplementary Fig. S9. To the best of our knowledge clusters **3a** and **3b** constitute the first pair of true isomers within the family of Se-protected NCs certified by X-ray crystallography. The Pd-Ag_ico_ average distances in **3a** and **3b** are equivalent (2.758(10) Å and 2.754(10) Å, respectively), as well as their average Ag_ico_-Ag_ico_ distance (2.901(9) Å and 2.896(10) Å, respectively). Thus, the structure of the Pd@Ag_12_ core in **3a** and **3b** is quite independent from the configuration of the outer sphere (Table 2). The average Ag_ico_@Se distance in both **3a** and **3b** are larger than the Ag_cap_@Se distances. The Se···Se bite distances in **3a** and **3b** are fairly similar (Table 2) and slightly shorter than those observed in [Ag_21_{Se_2_P(OEt)_2_}_12_]^20^ (3.697 Å) and [AuAg_20_{Se_2_P(OEt)_2_}_12_]^20^ (3.697 Å).

The two isomers assemble in a layer-by-layer mode. Each layer consists of pure **3a**(*T*) or **3b**(*C*_3_) (Fig. 6a). The *T* and *C*_3_ layer are alternately stacked along the [001] direction (Fig. 6b). The three-fold rotational axes of **3a** and **3b** are parallel to the *c* axis of the trigonal lattice. Finally, it is worth mentioning at this point that the isomer selectivity of the low temperature crystallization (**3**(*Pn*), *T* isomer) facilitates its further spectroscopic characterizations.

#### [PdAg_20_{Se_2_P(O^n^Pr)_2_}_12_], (4)

Given the synthesis of **2a**-**3** *via* ligand-exchange-induced structure transformation route it is indeed inevitable not to synthesize another normal propyl alkyl chain analog. Note that the precedence of structurally precise selenium protected alloy clusters is awfully inadequate. Thus, as shown in Fig. 1, we have endeavored the ligand replacement of dithiophosphates on **1** by diselenophosphates with linear alkyl chain (n-propyl). The reaction leads to the formation of [PdAg_20_{Se_2_P(O^n^Pr)_2_}_12_] (**4)** in 75 % yield. Its ^31^P{^1^H} spectrum in CDCl_3_ displays a signal at δ = 73.59 ppm flanked with two set of satellites (^1^*J*_P-Se_ = 604.51 and 710.40 Hz) at room temperature (Supplementary Data 1, Fig. 8). The ^1^H{^31^P} NMR spectrum of **4** in CDCl_3_ reveals three set of signals with multiplets ranged at δ = 4.03–4.02 ppm (corresponds to –OC*H*_*2*_ group), δ = 1.78–1.70 ppm (corresponds to –C*H*_2_) and at δ = 0.95–0.92 ppm (corresponds to –C*H*_3_) in an integration ratio of 1:1:1.5, which is clearly attributed to ^n^Pr group of di-propyl diselenophosphate ligands (Supplementary Data 1, Fig. S9). The ESI mass (positive-ion mode) spectrum shows a prominent band owing to [**4** + Ag]^+^ at *m*/*z* = 6056.02 (calcd. 6056.49), and its simulated isotopic distribution is in good agreement with the experimental one (Supplementary Fig. S10). Based on these spectroscopic evidences, the molecular structure of **4** should adopt the same *T* arrangement as that of **3a**. This is confirmed by the solid state structure of **4** obtained from single-crystal X-ray diffraction (Fig. 4 and Supplementary Fig. S11). Its structural metrics are similar to those of **3**(*Pn*) and **3a** (Table 2). Interestingly, **4** crystallize as a racemate in the *P*2_1_ space group.

**Fig. 8:**
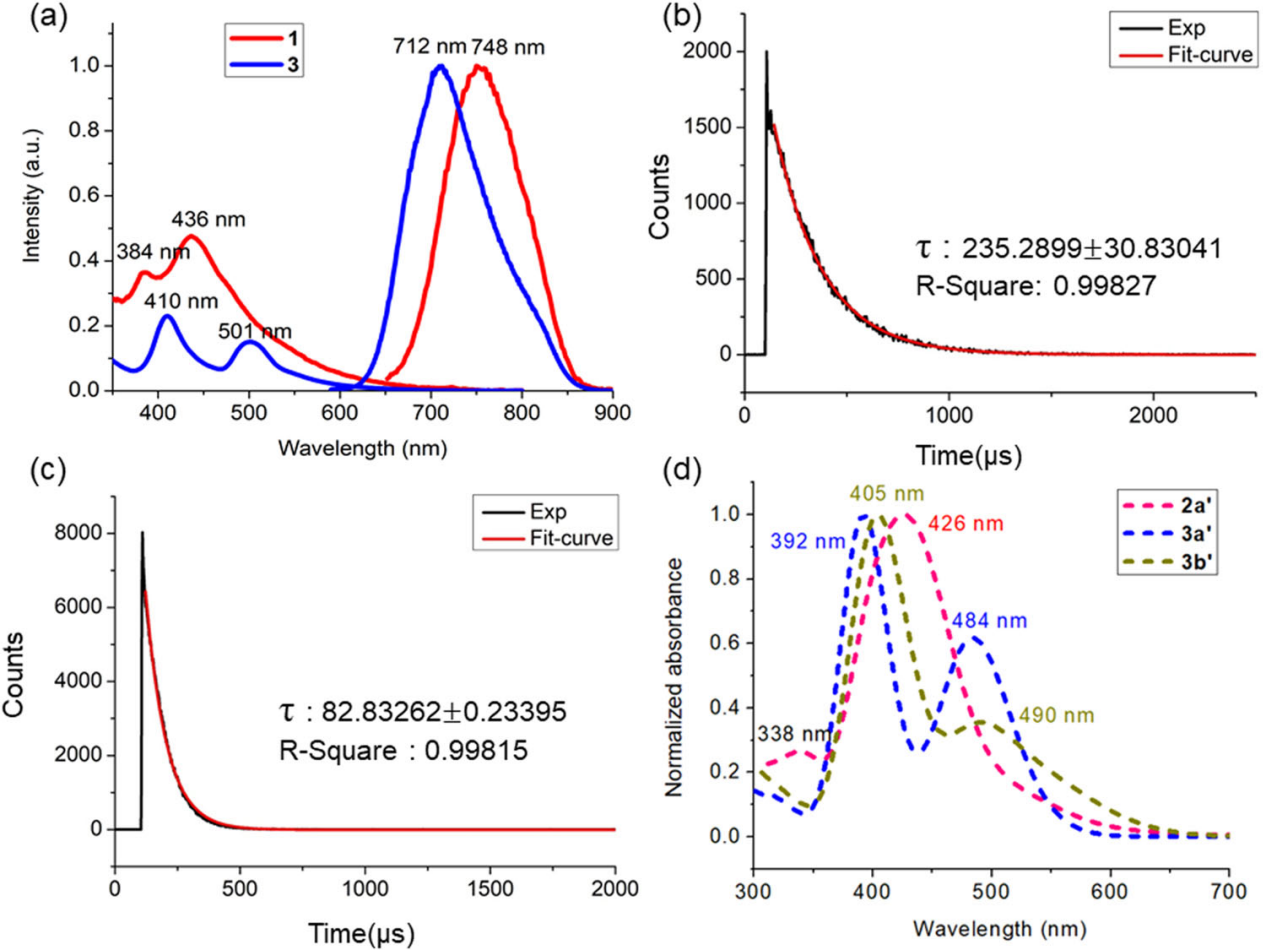
Experimental and theoretical photophysical studies of title clusters. **a** UV–vis spectrum of **1**, and **3** in 1 ×10^−5 ^M CH_2_Cl_2_ (left) and emission spectrum of **1** and **3** in MeTHF at 77 K (right). **b**, **c** Time-resolved photoluminescence spectrum of 2**a** and **3** at 77 K. **d** The TD-DFT-simulated UV–vis spectrum of **2a’, 3a’** and **3b’**.

#### Optical properties of the title NCs

It is interesting to note that the side chain in dithiophosph(in)ate ligands can lead to the variance of photoluminescence properties. The differed alkyl chains such as n-propyl (**1**), i-propyl (**2a**), i-butyl (**2b**), have least variance and look reddish while the phenyl derivative (**2c**) which was only obtained by ligand exchange appear to be orange to the naked eye. The UV–Vis spectra of **1**, **2a** and **2b** show similar broad optical absorption bands at 384 and 436 nm, the latter band being intense (Table 1, Fig. 7). On the other hand, the phenyl derivative **2c** features different absorption bands (419 and 486 nm) where the former is found to be more intense (Fig. 7). The absorption bands in **2c** are red-shifted to their alkyl relatives. The change from alkyl to phenyl of the dtp substituents can alter the photoluminescence intensity. Compounds **1**, **2a**, and **2b** show photoluminescence in their solution state at 77 K. Their emission maxima in 2-methyl tetrahydrofuran (MeTHF) occur at λ_max_ = 748 nm, 741 nm and λ_max_ = 732 nm, respectively (Fig. 7 and Supplementary Figs. S12 and S13). Cluster **2c** is also emissive in solution state at 77 K. Its emission maximum appears at 669 nm in MeTHF (Fig. 7 and Supplementary Fig. S13) which is blue shifted to its parent cluster **1**.

The UV–vis spectra feature two major broad absorption bands for **3** (λ_max_ = 410 and 501 nm) and **4** (λ_max_ = 408 and 498 nm) (Supplementary Fig. S14) which are red shifted with respect to those observed in their parent cluster **1** (λ_max_ = 384 and 436 nm) (Fig. 8a). Cluster **3** and **4** displays photoluminescence in solution at 77 K where the emission maximum in 2-methyl tetrahydrofuran occurs at 712 and 702 nm, respectively which are slightly blue shifted with respect to those of their dtp analogs **1** or **2a** (Fig. 8a and Supplementary Fig. S15). The time resolved photoluminescence spectra (77 K) of **2a–c, 3** and **4** exhibits a single exponential decay curve (Fig. 8b, c and Supplementary Figs. S16–18). The observed emission lifetimes (τ) of the dithiophosphate analogs **2a-b** (**2a**: τ = 235.3 µs and **2b**: τ = 198.8 µs) are comparatively longer than their diselenophosphate counterparts (**3**: τ = 82.8 µs and **4**: τ = 82.1 µs). Further the lifetime of that of dithiophosphinate analog **2c** is of 60 µs which is shorter compared to its dithiophosphate and diselenophosphate relatives. The emission lifetimes in the order of microseconds for NCs **2a–c, 3** and **4** indicate the occurrence of phosphorescence in each case.

#### Computational studies of title NCs

In a recent DFT investigation on the alloying of dichalcogenolate-protected Ag_21_ species^43^, we have shown that in the case of 8-electron NCs of the type [MAg_20_{dtp/dsep}_12_]^±q^ (M = group 9 to group 12 metal), when M is a group 9 or 10 metal, it strongly prefers occupying the center of the icosahedron, *i.e*., [M@Ag_20_{dtp/dsep}_12_]^±q^. The reason lies in the involvement of the nd(M) valence orbitals in the metal-metal bonding through their stabilization by the vacant superatomic 1D shell. Calculations on the *T*, *C*_3_ and *C*_1_^36^ structures of [M@Ag_20_{dtp/dsep}_12_]^±q^ indicated also a small energy differences between these structures, in particular between *T* and *C*_3_, independently from the nature of M. Calculations on the simplified model [PdAg_20_{Se_2_PH_2_}_12_] with a slightly different basis set as previously^35^ found the *T* isomer to be slightly more stable, both in total energy (ΔE = 3.7 kcal/mol) and free energy (ΔG = 0.2 kcal/mol), this last value being not significantly different from zero. Calculations on the less simplified model [PdAg_20_{Se_2_P(OMe)_2_}_12_] found similar results with ΔE = 3.7 kcal/mol and ΔG = 2.7 kcal/mol. Although calculations on the real clusters **3a** and **3b** were not performed owing to their large size, these results confirm our previous finding that the *T* and *C*_3_ structures are close in energy, with the *T* isomer tending to be slightly more favored in the case of diselenolate ligands. The very small computed energy difference between the two isomers is fully consistent with their observation as co-crystallized species. The *C*_1_ structure adopted by compound **2a** was also calculated in the case of the [PdAg_20_{Se_2_PH_2_}_12_] model. It was also found less stable than its *T* isomer (ΔE = 10.3 kcal/mol and ΔG = 4.8 kcal/mol). In the case of the dithiolate model [PdAg_20_{S_2_PH_2_}_12_], this energy difference is reduced (ΔE = 4.6 kcal/mol and ΔG = 0.0 kcal/mol), in agreement with the observation of **2a**. They illustrate close similarities in the bonding situation of the various isomers, in full consistency with their closeness in energy. All these computed species have their three highest occupied orbitals of 1P nature, whereas the 1D level correspond the lowest vacant orbitals.

TD-DFT calculations on [PdAg_20_{S_2_PH_2_}_12_] (*C*_1_) and [PdAg_20_{Se_2_PH_2_}_12_] (*T* and *C*_3_), as models for **2a**(**2a’**), **3a**(**3a’**) and **3b**(**3b’**), provided the simulated UV–Vis spectra shown in Fig. 8d. They are in good agreement with their experimental counterparts (Fig. 8d). The low-energy band is of 1P → 1D nature and a comparison of Figs. 8a and 8d let to suggest that the *T* isomer of **3** might be the dominant species in solution.

## Conclusions

In summary, we have isolated and characterized a series of dtp- and dsep-protected 8-electron superatomic Pd doped silver NCs, of which several were structurally characterized. These structurally precise NCs feature a Pd-centered Ag_12_ icosahedron capped by 8 silver(I) atoms and 12 dichalcogenolate ligands with metallic PdM_20_ frameworks of ideal *C*_2_, *T*_*h*_, and *D*_3_ symmetries, respectively, which reduce to *C*_1_, *T*, and *C*_3_, respectively, when the 12 ligands are considered. Selenium-ligand exchange on **1** (*C*_1_ symmetry) induced the formation of a pair of [PdAg_20_{Se_2_P(O^i^Pr)_2_}_12_] structural isomers that are *T*-symmetric (**3a**, *T* symmetry) and **3b** (*C*_3_ symmetry). To our knowledge this is the first ever reported isomeric pair of selenium-protected NCs existed in a unit cell. In fact, these are the rare evidence of structurally characterized alloy cluster completely covered by Se shell. Overall this work demonstrates that the ligand exchange synthetic method indeed provides insights to the development of both *pseudo* and true structural isomeric alloy NCs.

### Experimental methods

#### Reagents and Instrumentation

The reactions were carried out by using standard Schlenk techniques under N_2_ atmosphere. LiBH_4_ (2M in THF) and other chemicals were purchased from different commercial sources available and were used as received. The ligands and metal precursors used in this work, NH_4_[E_2_P(R)] (R = O^n^Pr, O^i^Pr, O^i^Bu, or Ph; E = S or Se)^44–46^, [Ag(CH_3_CN)_4_]PF_6_^47^ and Pd[S_2_P(O^n^R’)_2_]_2_ (R’ = O^n^Pr, O^i^Pr or O^i^Bu)^48^, were synthesized as described in the literature. All the solvents used in this work were distilled under N_2_ atmosphere. ESI-mass spectra were recorded on a Fison Quattro Bio-Q (Fisons Instruments, VG Biotech, U. K.). Bruker Advance DPX300 FT-NMR spectrometer was used to record NMR spectra that operate at 300 and 400 megahertz (MHz) while recording ^1^H, 121.49, and 161.9 MHz for ^31^P and 100 MHz for ^13^C. Residual solvent protons were used as a reference (*δ*, ppm, CDCl_3_, 7.26). ^31^P{^1^H} NMR spectra were referenced to external 85% H_3_PO_4_ at *δ* 0.00. UV–Visible absorption spectra were measured on a Perkin Elmer Lambda 750 spectrophotometer using quartz cells with path length of 1 cm. The elemental analyses were done using a Perkin-Elmer 2400 CHN analyzer. Photoluminescence spectra and lifetime measurements were carried out using an Edinburgh FLS920 fluorescence spectrometer.

### Synthesis of [PdAg_20_{S_2_P(O^i^Pr)_2_}_12_] (2a)

#### Ligand exchange method

In an oven-dried Schlenk tube, [PdAg_20_{S_2_P(O^n^Pr)_2_}_12_], **1** (0.100 g, 0.0207 mmol) was dissolved in THF (5 mL) and was placed at −20 °C for 15 min. Then twelve equivalents of NH_4_[S_2_P(O^i^Pr)_2_] (0.057 g, 0.2484 mmol) were added to the solution. The reaction mixture was stirred for 1 h in the same temperature. The solvent was dried under vacuum and the obtained residue was extracted with hexane (3×5 mL) and filtered to remove decomposed impurities from ligand. In this reaction NH_4_[S_2_P(O^n^Pr)_2_] has been isolated as byproduct. The hexane solution was passed through Al_2_O_3_ column followed by ether. The brown-red solution obtained was dried which yielded (0.070 g, 70.17% based on Pd) [PdAg_20_{S_2_P(O^i^Pr)_2_}_12_] (**2a**).

- *Direct method.* In an oven-dried Schlenk flask, [Ag(CH_3_CN)_4_](PF_6_) (0.5 g, 1.2 mmol) was suspended in THF (15 mL). To this NH_4_[S_2_P(O^i^Pr)_2_] (0.14 g, 0.6 mmol), and [Pd{S_2_P(O^i^Pr)_2_}] (0.020 g, 0.0626 mmol) were added sequentially. Then the reaction flask was placed in a low temperature bath at −20 °C for 15 min. LiBH_4_ ∙ THF (0.2 mL, 0.8 mmol) was added slowly via syringe to the reaction mixture and the orange colored solution immediately turned to black after the LiBH_4_ ∙ THF addition. The reaction was aged 24 h at the same temperature. The solvent was evaporated under vacuum. In order to eliminate decomposed impurities from ligand, the residue was thoroughly washed with deionized water and was subsequently extracted in CH_2_Cl_2_. The resulting CH_2_Cl_2_ solution was dried and then the residue was further dissolved in hexane. Then solution in hexane was passed through the Al_2_O_3_ column. Then the column was run by hexane/ether (80:20 *v/v*) mixture which resulted to brown-red solid. The brown-red solid was then re-dissolved in CH_2_Cl_2_ and was chromatographed on silica gel TLC plates. Elution with a hexane/CH_2_Cl_2_ (40:60 *v/v*) mixture which yielded molecularly pure compound (**2a**) in 41 % yield (0.124 g based on Pd).

**2a**. ESI-MS(*m/z*) [M + Ag]^+^ calcd. for C_72_H_168_Ag_21_O_24_P_12_PdS_24_, 4930.97; found, 4931.15; ^1^H NMR (22 °C, 300 MHz, CDCl_3_, *δ*, ppm): 4.97–4.85 (m, 24H, OC*H*), 1.35–1.33 (d, 144 H, C*H*_3_); ^31^P{^1^H} NMR (22 °C, 121.49 MHz, CDCl_3_, δ, ppm): 101.66; UV–vis [λ_max_ in nm, (ε in M^−1^cm^−1^)]: 384 (71254), 436 (106058).

### Synthesis of [PdAg_20_{S_2_P(O^i^Bu)_2_}_12_] (2b)

- *Direct method.* In an oven-dried Schlenk flask, [Ag(CH_3_CN)_4_](PF_6_) (0.5 g, 1.2 mmol) was suspended in THF (15 mL). To this NH_4_[S_2_P(O^i^Bu)_2_] (0.155 g, 0.6 mmol), and [Pd{S_2_P(O^i^Bu)_2_}] (0.030 g, 0.086 mmol) were added one after another. Then the reaction flask was placed in low temperature bath at −20 °C for 15 min. LiBH_4_ ∙ THF (0.2 mL, 0.8 mmol) was added slowly via syringe to the reaction mixture and then the resulting solution was stirred at the same temperature for 24 h. The orange colored solution instantaneously turned to black after the addition of LiBH_4_ ∙ THF. The solvent was dried completely under vacuum. Then the residue was washed thoroughly with deionized water followed by the extraction in CH_2_Cl_2_. The resulting CH_2_Cl_2_ solution was dried and then the residue was further dissolved in hexane. That solution in hexane was passed through the Al_2_O_3_ column. Then the column was run by hexane/ether (80:20 *v/v*) mixture which resulted to red solid. Moreover the red solid was re-dissolved in CH_2_Cl_2_ and was chromatographed on silica gel TLC plates. Elution with a hexane/CH_2_Cl_2_ (40:60 *v/v*) mixture which yielded molecularly pure compound (**2b**) in 40 % yield (0.177 g based on Pd). Compound **2b** can also be synthesized via ligand replacement method but the crystalline materials can only be obtained by a co-reduction method. Note that in this metiod, compound **1** was treated with twelve equivalents of NH_4_[S_2_P(O^i^Bu)_2_] in THF at ambient temperature for 10 min.

**2b**. ESI-MS(*m/z*) [M + Ag]^+^ calcd. for C_96_H_216_Ag_21_O_24_P_12_PdS_24_, 5267.62; found, 5267.64; ^1^H NMR (22 °C, 300 MHz, CDCl_3_, *δ*, ppm). 3.91 (t, *J* = 6.76 Hz, 48H), 2.00 (m, *J* = 6.08 Hz, 24H), 0.94 (d, *J* = 6.12 Hz, 144H); ^31^P{^1^H} NMR (22 °C, 161.9 MHz, CDCl_3_, δ, ppm); 103.85 UV–vis [λ_max_ in nm, (ε in M^−1^cm^−1^)]: 384 (25994), 436 (47813).

### Synthesis of [PdAg_20_{S_2_PPh_2_}_12_] (2c)

#### Ligand exchange method

In an oven-dried Schlenk tube, [PdAg_20_{S_2_P(O^n^Pr)_2_}_12_], **1** (0.025 g, 0.005 mmol) was dissolved in THF (10 mL) and was placed at −20 °C for 5 min. Then K[S_2_PPh_2_] (0.019 g, 0.065 mmol) was added to the solution. The resulting reaction mixture was allowed to stir for 2 min at the same temperature. The solvent was then evaporated under vacuum. The obtained residue was extracted with hexane (3 × 5 mL) and filtered to eliminate by-product NH_4_[S_2_P(O^n^Pr)_2_]. The hexane solution was passed through Al_2_O_3_ column followed by ether. Then the pure orange compound (**2c**) obtained was dried which produced 65 % yield (0.017 g based on Pd).

**2c**. ESI-MS(*m/z*) [M + 2Ag]^2+^ calcd. for C_144_H_120_Ag_20_P_12_PdS_24_, 2735.89; found, 2735.76; ^1^H NMR (22 °C, 300 MHz, CDCl_3_, *δ*, ppm). 7.44, 7.85(m,120 H, C_6_H_5_); ^31^P{^1^H} NMR (22 °C, 121.49 MHz, CDCl_3_, δ, ppm): 63.14; UV–vis [λ_max_ in nm, (ε in M^−1^cm^−1^)]: 419 (44872), 486 (28290).

### Synthesis of [PdAg_20_{Se_2_P(O^i^Pr)_2_}_12_] (3, 3a and 3b)

#### Synthesis of 3a and 3b

In a Flame-dried Schlenk tube, [PdAg_20_{S_2_P(O^n^Pr)_2_}_12_], **1** (0.100 g, 0.0207 mmol) was dissolved in THF (5 mL) and was placed at −20 °C for 15 min. Twelve equivalents of NH_4_[Se_2_P(O^i^Pr)_2_] (0.080 g, 0.2484 mmol) was added to that solution. The resulting mixture was stirred for 2 min in the same temperature. The solvent was dried completely under vacuum and residue was extracted in hexane (3×5 mL) and filtered to remove decomposed impurities from ligand. In this reaction NH_4_[S_2_P(O^n^Pr)_2_] has been isolated as byproduct. The solution was passed through Al_2_O_3_ followed by the addition acetone resulted the wine-red colored solution. Then the solution was then evaporated for further analysis. Suitable single crystals for X-ray diffraction were grown at low and ambient temperatures. The crystallization in the ambient temperature revealed a co-crystallization of **3a** and **3b** with chemical formula [PdAg_20_{Se_2_P(O^i^Pr)_2_}_12_]. The yield of **3a·3b** was 78 % (0.096 g based on Pd).

**3a**. ESI-MS(*m/z*) [M + Ag]^+^ calcd. for C_72_H_168_Ag_21_O_24_P_12_PdSe_24_, 6056.48; found 6055.77; ^1^H NMR (22 °C, 400 MHz, *d*_6_-Acetone, δ, ppm): 5.07–4.95 (q, 24H, OCH), 1.47–1.38 (m, 144H, CH_2_); ^31^P{^1^H} NMR (22 °C, 161. 9 MHz, CDCl_3_, δ, ppm): 68.16 (J_*P-Se*_ = 604.51 and 710.40 Hz; UV–vis [λ_max_ in nm, (ε in M^−1^cm^−1^)]: 410 (1033762), 501 (690654).

#### Alternative synthesis of 3 or 3a

In a Flame-dried Schlenk tube, [PdAg_20_{S_2_P(O^i^Pr)_2_}_12_], **2a** (0.100 g, 0.0207 mmol) was dissolved in THF (5 mL) and was placed at −20 °C for 15 min. Then twelve equivalents of NH_4_[Se_2_P(O^i^Pr)_2_] (0.080 g, 0.2484 mmol) was added to the solution. The resulting mixture was stirred for 2 min at the same temperature. The solvent was dried under vacuum and residue was then extracted with hexane (3 × 5 mL) and filtered to remove decomposed impurities from ligand. In this reaction NH_4_[S_2_P(O^n^Pr)_2_] has been isolated as byproduct. The hexane solution was passed through Al_2_O_3_ column followed by acetone which exclusively led to the isolation of compound **3** in 73% yields (0.090 g based on Pd).

**3 or 3a**. ESI-MS(*m/z*) [M + Ag]^+^ calcd for C_72_H_168_Ag_21_O_24_P_12_PdSe_24_, 6056.48; found 6055.77; ^1^H NMR (22 °C, 400 MHz, *d*_6_-Acetone, δ, ppm): 5.07–4.95 (q, 24H, OCH), 1.47–1.38 (m, 144H, CH_2_); ^31^P{^1^H} NMR (22 °C, 161. 9 MHz, CDCl_3_, δ, ppm): 68.16 (J_*P-Se*_ = 604.51 and 710.40 Hz); UV–vis [λ_max_ in nm, (ε in M^−1^cm^−1^)]: 410 (1033762), 501 (690654).

#### Synthesis of [PdAg_20_{Se_2_P(O^n^Pr)_2_}_12_] (4)

In an oven-dried Schlenk tube, [PdAg_20_{S_2_P(O^n^Pr)_2_}_12_], **1** (0.025 g, 0.005 mmol) was dissolved in THF (5 mL) and was placed at −20 °C for 15 min. Then twelve equivalents of NH_4_[Se_2_P(O^n^Pr)_2_] (0.021 g, 0.065 mmol) was added to the solution. The resulting mixture was stirred for 2 min in the same temperature. The solvent was evaporated under vacuum and residue was extracted with hexane (3 × 5 mL) and filtered to remove decomposed impurities from ligand. In this reaction NH_4_[S_2_P(O^n^Pr)_2_] has been isolated as byproduct. The hexane solution was passed through Al_2_O_3_ column followed by acetone which exclusively led to the isolation of [PdAg_20_{Se_2_P(O^n^Pr)_2_}_12_] (**4**) in 75% yields (0.022 g based on Pd).

**4**: ESI-MS(*m/z*) [M + Ag]^+^ calcd for C_72_H_168_Ag_21_O_24_P_12_PdSe_24_, 6056.48; found 6056.02; ^1^H NMR (22 °C, 400 MHz, *d*_6_-acetone, δ, ppm): 0.90 (t, 72H, C*H*_3_), 1.59 (q, 48H, C*H*_2_), 3.85(m, 48, C*H*_2_); ^31^P{^1^H} NMR (22 °C, 161.9 MHz, *d*_6_-acetone, δ, ppm): 73.59 (^1^*J*_P-Se_ = 604.51 and 710.40 Hz); UV–vis [λ_max_ in nm, (ε in M^−1^cm^−1^)]: 408(95700), 498(68700).

#### Single crystal X-ray structure determination

Single crystals suitable for X-ray diffraction analysis of **2a, 2b**, **3, (3a** and **3b**) and **4** were obtained by diffusing hexane into concentrated CH_2_Cl_2_ or acetone solution at room and (or) low temperature within one or two weeks. The single crystals were mounted on the tip of glass fiber coated in paratone oil, and then frozen. Data were collected on a Bruker APEX II CCD diffractometer using graphite monochromated Mo K*α* radiation (*λ* = 0.71073 Å) at 150 K (**2a**, **3**, (**3a** and **3**b)) and 100 K (**2b** and **4**). Absorption corrections for area detector were performed with SADABS^49^ and the integration of raw data frame was performed with SAINT^50^. The structure was solved by direct methods and refined by least-squares against *F*^2^ using the SHELXL-2018/3 package^51,52^, incorporated in SHELXTL/PC V6.14^53^. All non-hydrogen atoms were refined anisotropically. The compound **2a** was crystallized in P$\bar{1}$ space group; one of the isopropyl groups (C4-C6) was disordered over two positions with the same occupancy. The compound **3** is crystallized in *Pn* space group and one of the isopropyl groups (C16-C18) was disordered over two positions, refined with the same occupancy; **3a** and **3b** were co-crystallized in *P*3_1_*c* space group where two Se atoms (Se9 and Se14) of the ligands were disordered over two positions with 90% and 10% occupancy and one of the isopropyl groups (C13-C15) was disordered over two positions with the same occupancy. Crystallographic data for compounds **2a-b**, **3**, **3a-b**, and **4** have been listed below in Table 3. The X-ray crystallographic coordinates for structures reported in this Article have been deposited at the Cambridge Crystallographic Data Center (CCDC), under deposition number CCDC 1985874 (**2a**), CCDC 2189558 (**2b**), CCDC 1985873 (**3**), CCDC 1985872 (**3a-b**), and CCDC 1985875 (**4**). These data can be obtained free of charge from The Cambridge Crystallographic Data Center via www.ccdc.cam.ac.uk/data_request/cif.” The crystallographic information files for compounds **2a-b**, **3**, **3a-b**, and **4** are provided as Supplementary Data 2.

**Table 3:** Crystallographic data for compounds **2a-b**, **3**, **3a-b**, and **4**.

Compound	2a	2b	3	3a and 3b	4
Empirical formula	C_72_H_168_Ag_20_O_24_P_12_PdS_24_	C_96_H_216_Ag_20_O_24_P_12_Pd S_24_	C_72_H_168_Ag_20_O_24_P_12_Pd Se_24_	C_72_H_168_Ag_20_O_24_P_12_PdSe_24_	C_72_H_168_Ag_20_O_24_P_12_PdSe_24_
Crystal system, space group	Triclinic, *P*$\bar{1}$	Hexagonal, *P* 6_3_	Monoclinic, *P*n	Trigonal, *P*3_1_c	Monoclinic, *P*2_1_
*a* (Å)	15.8599(12)	19.2447(7)	17.9414(11)	22.5252(9)	15.9494(6)
*b* (Å)	18.0946(14)	19.2447(7)	26.7432(15)	22.5252(9)	29.3080(12)
*c* (Å)	29.382(2)	26.1772(12)	18.8174(11)	40.401(2)	16.4792(7)
*α* (°)	82.1487(15)	90	90	90	90
*β* (°)	77.2002(15)	90	113.2950(10)	90	107.4809(10)
*γ* (°)	66.2229(13)	120	90	120	90
V(Å^3^)	7514.0(10)	8396.1(7)	8292.8(8)	17752.5(17)	7347.4(5)
Z	2	2	2	4	2
ρ_calcd_, g·cm^−3^	2.132	2.041	2.382	2.226	2.689
μ, mm^−1^	3.166	2.841	7.834	7.319	8.842
Temperature, K	150(2)	100(2)	150(2)	150(2)	100(2)
θ_max_, deg./completeness, %	25.00/95.8	24.999/100	25.00/99.6	24.99/99.9	24.99 / 99.9
Reflections collected/unique	44,057/25,382 [R(int) = 0.0237]	58,687/7962 [R(int) = 0.1157]	48,427/22,452 [R(int) = 0.0217]	113,818/ 20,789 [R(int) = 0.0863]	48,724/25,686 [R(int) = 0.0512]
Restraints/parameters	459/1432	582/680	818/1550	712/967	456/1440
R1^a^, wR2^b^ [I > 2σ(I)]	0.0668,0.1355	0.0798, 0.1909	0.0342, 0.0887	0.1125,0.3046	0.0399,0.0971
R1^a^, wR2^b^ (all data)	0.0867,0.1511	0.1036, 0.2320	0.0387, 0.0919	0.1291,0.3191	0.0477,0.1046
Absolute structure parameter	–	0.40(18)	0.037(9)	0.18(3)	0.327(9)
Goodness of fit on *F*^2^	1.084	1.102	1.031	1.037	1.023
Largest diff. peak and hole, e/Å ^3^	1.897 and −1.680	1.686 and −1.393	1.572 and −0.866	2.540 and −2.782	1.750 and −1.625

^a^$$R1=\Sigma ||{F}_{o}|-|{F}_{c}||/\Sigma |{F}_{o}|$$.

^b^$$wR2={\{\Sigma [w{({F}_{o}^{2}-{{{{{{\rm{F}}}}}}}_{c}^{2})}^{2}]/\Sigma [w{({F}_{o}^{2})}_{2}]\}}^{1/2}$$.

#### Computational details

DFT calculations were carried out on simplified model clusters with the Gaussian 16 package^54^. The considered ligand simplifications allow to save a huge amount of CPU time and its validity has been proven in many occasions^20–22,36,41,43^. The BP86 functional^55,56^ was used together with the general triple-ζ polarized Def2-TZVP basis set from EMSL Basis Set Exchange Library^57,58^. All the optimized geometries were ascertained as true minima on the potential energy surface by performing vibrational frequency calculations. The natural atomic orbital (NAO) charges were computed with the NBO 6.0 program^59^. The UV–visible transitions were calculated on the above-mentioned optimized geometries by means of time-dependent DFT (TD-DFT) calculations, with the CAM-B3LYP functional^60^ and the LanL2DZ + pol^61–63^ basis set. The UV–visible spectra were simulated from the computed TD-DFT transitions and their oscillator strengths by using the SWizard code^64^, each transition being associated with a Gaussian function of half-height width equal to 1000 cm^−1^. The Cartesian coordinates for computed compounds are provided as Supplementary Data 3.

## Supplementary information

Supplementary Information

Description of Additional Supplementary Files

Supplementary data 1

Supplementary data 2

Supplementary data 3

## Data Availability

Supplementary Information contains ESI-MS, X-ray crystallographic data analysis Figures, computational data and photophysical studies of title compounds. NMR spectra of all the compounds are available in Supplementary Data 1. Crystal structures of compounds are provided in Supplementary Data 2. The Cartesian coordinates calculated for all the model compounds are given in Supplementary Data 3. The authors declare that all the relevant data belongs to the findings of this study are available within the article and its supplementary information files. They are also from the author (C.W.L.) upon reasonable request.
