# Supplementary material for: Surface modifications of eight-electron palladium silver superatomic alloys
Source: Commun Chem. 2022 Nov 19;5:151. doi: 10.1038/s42004-022-00769-2 (PMC9814913; doi:10.1038/s42004-022-00769-2)
Supplement: Supplementary file 1 — Supplementary Information [file 42004_2022_769_MOESM1_ESM.pdf]

Supplementary Information

Surface modifications of eight-electron palladium silver superatomic alloys

Subrat Kumar Barik[1,3,4], Chih-Yuan Chen[1], Tzu-Hao Chiu[1], Yu-Rong Ni[1], Franck Gam,[2] Isaac Chantrenne,[2] Samia Kahlal,[2] Jean-Yves Saillard,[2]* C. W. Liu [1]*

[1]Department of Chemistry, National Dong Hwa University, No. 1, Sec. 2, Da Hsueh Rd. Shoufeng, Hualien 97401, Taiwan (Republic of China). email:chenwei@mail.ndhu.edu.tw

[2]CNRS, ISCR-UMR 6226, Univ Rennes, Rennes F-35000, France.

[3]Department of Chemistry, Veer Surendra Sai University of Technology, Burla, Sambalpur, Odisha, 768018, India.

[4]Department of Chemistry, C. V. Raman Gobal University, Bidya Nagar, Bhubaneswar, Odisha, 752054, India

Table of Contents

Entry	page number
**Supplementary Table**	S5

Supplementary Table S1. A summary of reported M@Ag_{20} (M = Pt or Pd) superatomic alloy nanoclusters based on M_{21} cluster template.

Supplementary Figures S6-S18

Supplementary Figure S1. ESI-MS (Positive mode) of [**2b**+Ag]$^{+}$. Insets: experimental (black) and simulated (blue) mass spectra.

Supplementary Figure S2. ESI-MS (Positive mode) of [**2c**+2Ag]$^{2+}$. Insets: experimental (blue) and simulated (red) mass spectra.

Supplementary Figure S3. (a) $^{31}P\{^{1}H\}$ (121.49 MHz, $CDCl_3$) NMR spectrum of **1** before the reaction. (b) $^{31}P\{^{1}H\}$ (121.49 MHz, $CDCl_3$) NMR spectrum of reaction mixture after the removal of byproduct $NH_4[S_2P(O^nPr)_2]$ in 15 minutes. (c) $^{31}P\{^{1}H\}$ (121.49 MHz, $CDCl_3$) NMR spectrum of **2a** after purification of reaction mixture.

Supplementary Figure S4. (a) Illustration of pseudo-C_2 symmetry in the Pd@Ag_{20} core in **1**; (colour code. Pd: orange; Ag_{ico}: pink, and Ag_{cap}: blue, red, and gray). (b) Illustration of *pseudo*-C_2 symmetry in the Pd@Ag_{20} core in **2a**; (colour code. Pd: orange; Ag_{ico}: pink, and Ag_{cap}: blue, red, and gray). (c) Illustration of *pseudo*-D_3 symmetry in the Pd@Ag_{20} core in **2b**; (colour code. Pd: orange; Ag_{ico}: pink, and Ag_{cap}: blue, red, green and gray).

Supplementary Figure S5. Diffrent type of coordination modes of di-isopropyl dithiophosphate (dtp) ligands in **2a** (isopropyl groups omitted for clarity).

Supplementary Figure S6. Diffrent type of coordination modes of di-isopropyl dithiophosphate (dtp) ligands in **2b** (isopropyl groups omitted for clarity).

Supplementary Figure S7. Naked eye picture of ligand exchange reaction on to compound **1.**

Supplementary Figure S8. Positive ESI-MS spectrum of **3**. The inset shows the experimental (top) and theoretical spectra (bottom).

Supplementary Figure S9. (a) Illustration of transformation of $[PdAg_{20}(dsep)_{12}]$ NC with *T* symmetry (**3a**) to C_3 symmetry (**3b**); Out of twelve, nine dsep ligands are shown in orange. The remaining three dsep ligands are mentioned in red, cyan, and pink colour in order to elucidate their change in position in C_3 symmetry in **3b**. (Colour code. Pd: blue green; Ag: Gray). (b) Transformation of $[PdAg_{20}]$ core with T symmetry in **3a** to C_3 symmetry in **3b**.

Supplementary Figure S10. Positive ESI-MS spectrum of **4**. The inset shows the experimental (blue) and theoretical spectra (orange).

Supplementary Figure S11. (a) Total structure of $[PdAg_{20}\{Se_2P(O^iPr)_2\}_{12}]$ (**3**) (isopropoxy groups are shown in capped sticks model), (b) Illustration of Pd@Ag_{20} metallic core in **3** with T_h symmetry, (c) Total structure of $[PdAg_{20}\{Se_2P(O^nPr)_2\}_{12}]$ (**4**) (propoxy groups are shown in capped sticks model), (d) Illustration of Pd@Ag_{20} metallic core in **4** with T_h symmetry, (Colour code. Pd: light green; Ag_{ico}: blue, Ag_{cap}: pink; Se: orange; P: green).

Supplementary Figure S12. UV-vis, excitation and emission spectra of compound **2b** in MeTHF.

Supplementary Figure S13. Excitation (left) and emission (right) spectra of compound **2c** in MeTHF at 77K.

Supplementary Figure S14. UV–vis spectrum of **4** in 1 x 10^{-5} M CH_2Cl_2.

Supplementary Figure S15. Excitation and emission spectra of compounds **4** in MeTHF at 77K.

Supplementary Figure S16. Time-resolved photoluminescence spectrum of **2b** at 77K.

Supplementary Figure S17. Time-resolved photoluminescence spectrum of **2c** at 77K.

Supplementary Figure S18. Time-resolved photoluminescence spectrum of **4** at 77K.

Supplementary Table S1. A summary of reported $M@Ag_{20}$ (M = Pt or Pd) superatomic alloy nanoclusters based on M_{21} cluster template.

Cluster composition[a]	Synthetic method	Focus	Ref.[a]
$[PtAg_{20}\{S_2P(O^nPr)_2\}_{12}]$	Solution phase co-reduction	Crystal structure	[35]
$[PtAg_{20}\{Se_2P(O^iPr)_2\}_{12}]$	Ligand exchange	Crystal structure	[21]
$[PtAg_{20}\{Se_2P(O^nPr)_2\}_{12}]$	Ligand exchange	Crystal structure	[21]
$[PtAg_{20}\{Se_2P(CH_2CH_2Ph)_2\}_{12}]$	Ligand exchange	Crystal structure	[21]
$[PdAg_{20}\{S_2P(O^nPr)_2\}_{12}]$ (**1**)	solution phase co-\reduction	Crystal structure	[36]
$[PdAg_{20}\{S_2P(O^iPr)_2\}_{12}]$ (**2a**)	solution phase co-reduction and Ligand exchange	Crystal structure	**this work**
$[PdAg_{20}\{S_2P(O^iBu)_2\}_{12}]$ (**2b**)	solution phase co-reduction	Crystal structure	**this work**
$[PdAg_{20}\{S_2PPh_2\}_{12}]$ (**2c**)	Ligand exchange	ESI MS	**this work**
$[PdAg_{20}\{Se_2P(O^iPr)_2\}_{12}]$ (**3**)	Ligand exchange	Crystal structure	**this work**
$[PdAg_{20}\{Se_2P(O^nPr)_2\}_{12}]$ (**4**)	Ligand exchange	Crystal structure	**this work**

[a] corresponds to the respective references which are cited in the main manuscript in references section.

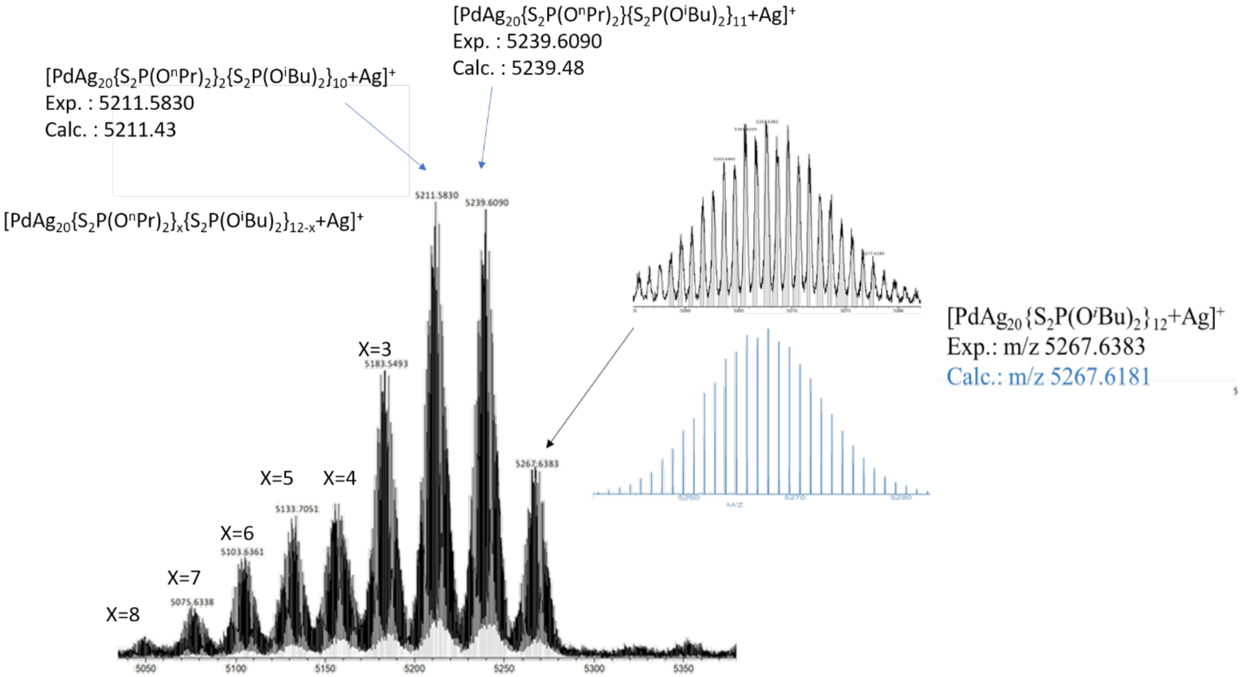

Supplementary Figure S1. ESI-MS (Positive mode) of [**2b**+Ag]$^+$. Insets: experimental (black) and simulated (blue) mass spectra.

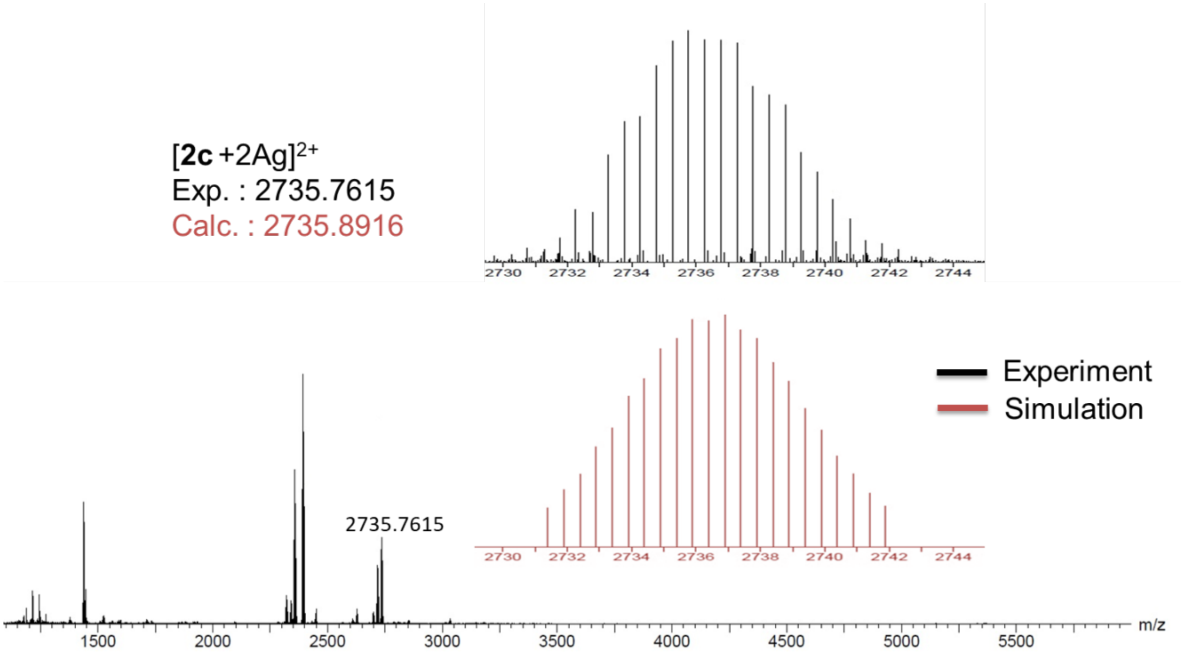

Supplementary Figure S2. ESI-MS (Positive mode) of [**2c**+2Ag]$^{2+}$. Insets: experimental (black) and simulated (red) mass spectra.

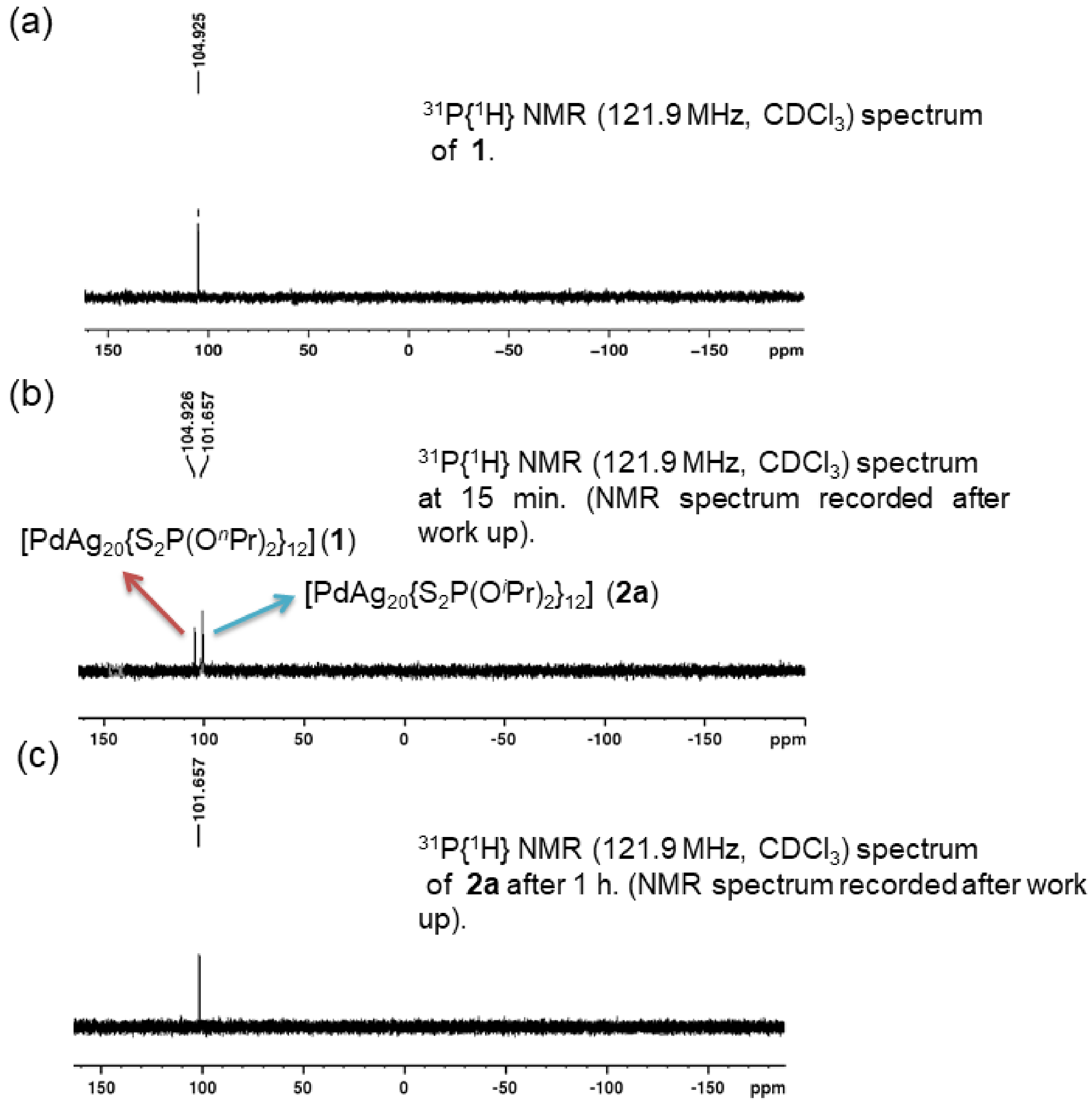

Supplementary Figure S3. (a) $^{31}P\{^1H\}$ (121.49 MHz, $CDCl_3$) NMR spectrum of **1** before the reaction. (b) $^{31}P\{^1H\}$ (121.49 MHz, $CDCl_3$) NMR spectrum of reaction mixture after the removal of byproduct $NH_4[S_2P(O^nPr)_2]$ in 15 minutes. (c) $^{31}P\{^1H\}$ (121.49 MHz, $CDCl_3$) NMR spectrum of **2a** after purification of reaction mixture.

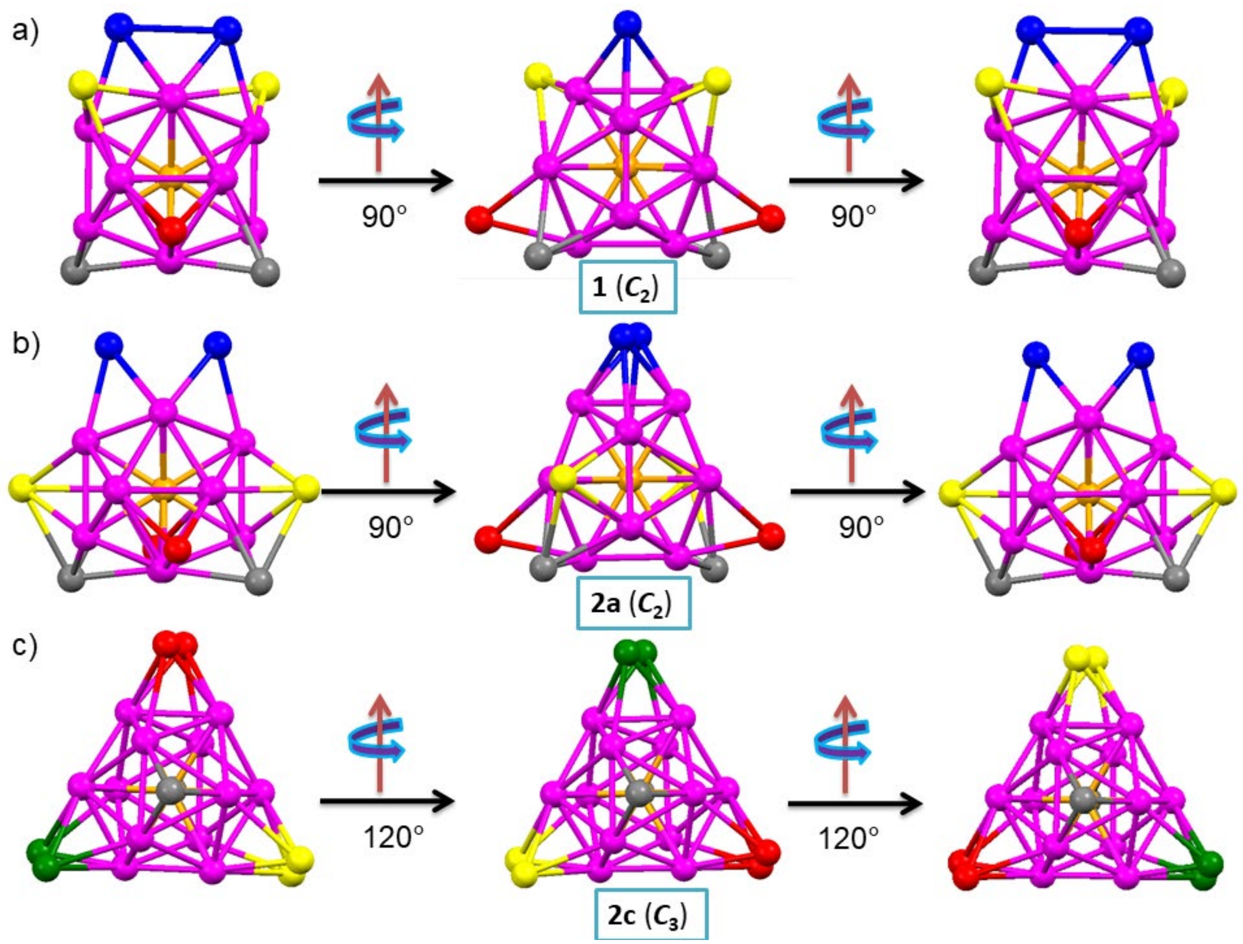

Supplementary Figure S4. (a) Illustration of pseudo-C_2 symmetry in the Pd@Ag_{20} core in **1**; (colour code. Pd: orange; Ag_{ico}: pink, and Ag_{cap}: blue, red, and gray). (b) Illustration of *pseudo*-C_2 symmetry in the Pd@Ag_{20} core in **2a**; (colour code. Pd: orange; Ag_{ico}: pink, and Ag_{cap}: blue, red, and gray). (c) Illustration of *pseudo*- D_3 symmetry in the Pd@Ag_{20} core in **2b**; (colour code. Pd: orange; Ag_{ico}: pink, and Ag_{cap}: blue, red, green and gray).

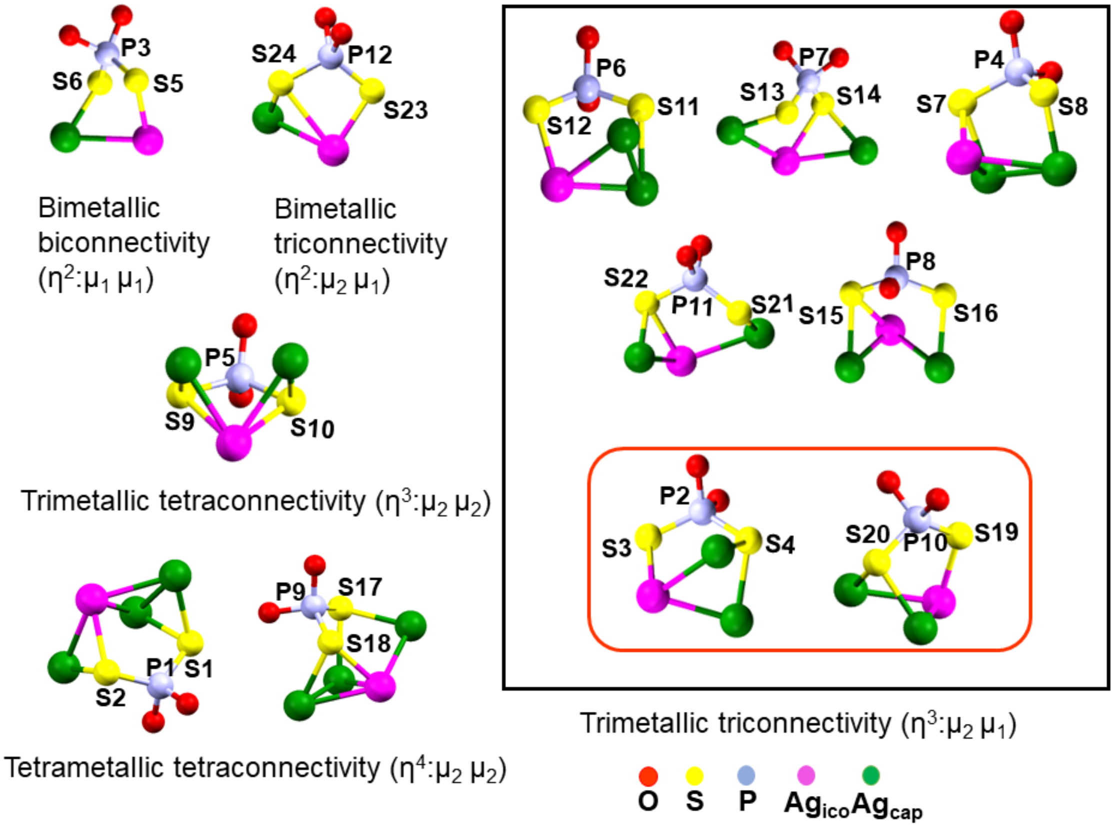

Supplementary Figure S5. Diffrent type of coordination modes of di-isopropyl dithiophosphate (dtp) ligands in **2a** (isopropyl groups omitted for clarity).

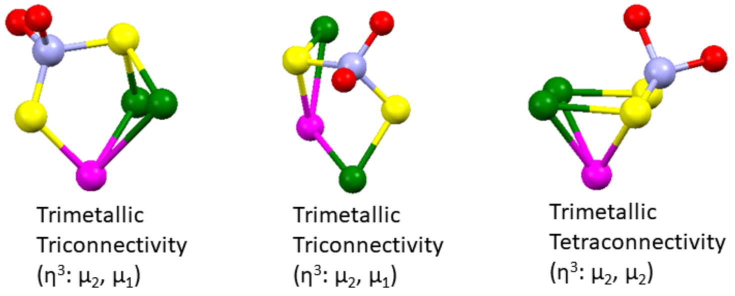

Supplementary Figure S6. Diffrent type of coordination modes of di-isopropyl dithiophosphate (dtp) ligands in **2b** (isopropyl groups omitted for clarity).

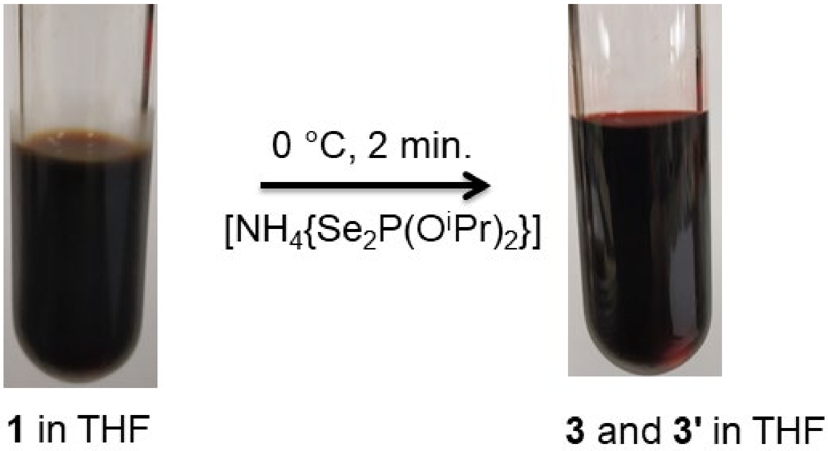

Supplementary Figure S7. Naked eye picture of ligand exchange reaction on to compound **1.**

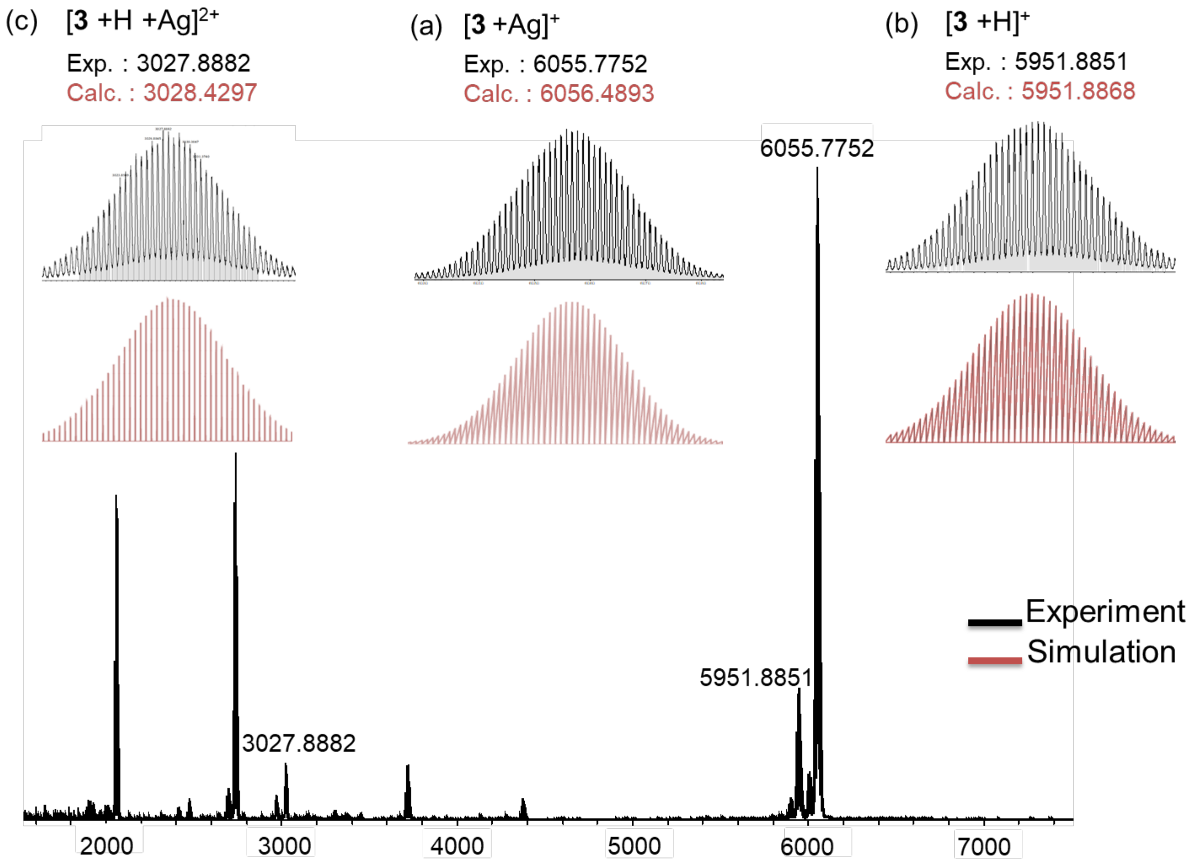

Supplementary Figure S8. Positive ESI-MS spectrum of **3**. The inset shows the experimental (top) and theoretical spectra (bottom).

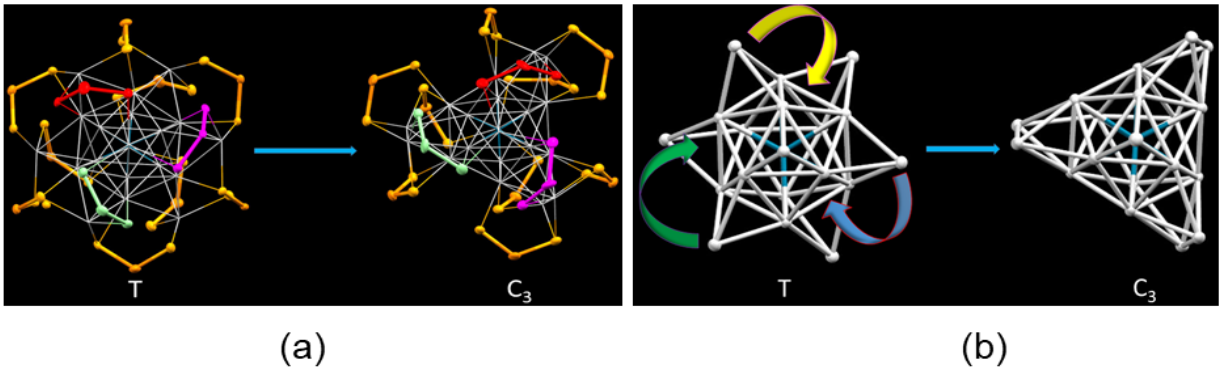

Supplementary Figure S9. (a) Illustration of transformation of [$PdAg_{20}(dsep)_{12}$] NC with *T* symmetry (**3a)** to C_3 symmetry (**3b**); Out of twelve, nine dsep ligands are shown in orange. The

remaining three dsep ligands are mentioned in red, cyan, and pink colour in order to elucidate their change in position in C_3 symmetry in **3b**. (Colour code. Pd: blue green; Ag: Gray). (b) Transformation of [$PdAg_{20}$] core with T symmetry in **3** to C_3 symmetry in **3b**.

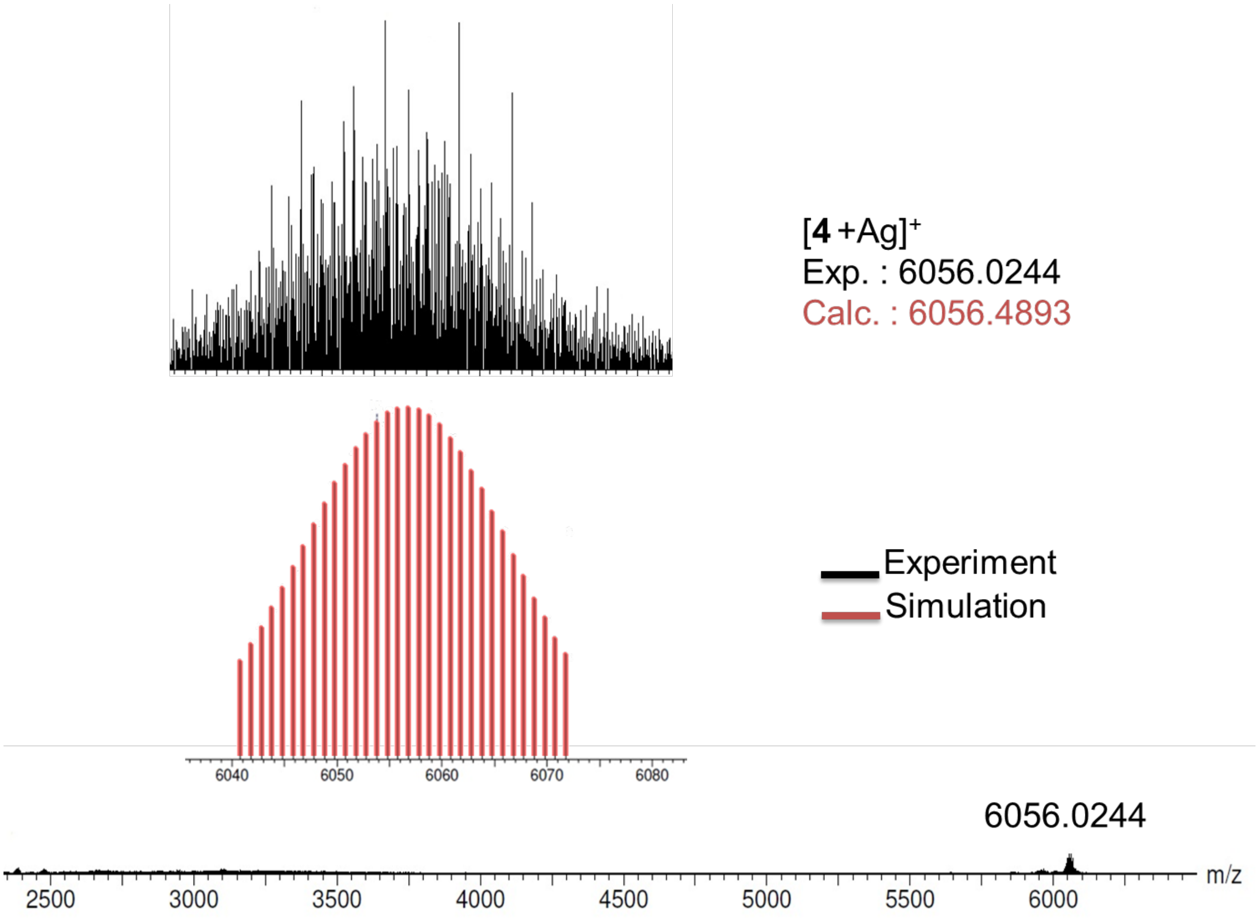

Supplementary Figure S10. Positive ESI-MS spectrum of **4**. The inset shows the experimental (blue) and theoretical spectra (red).

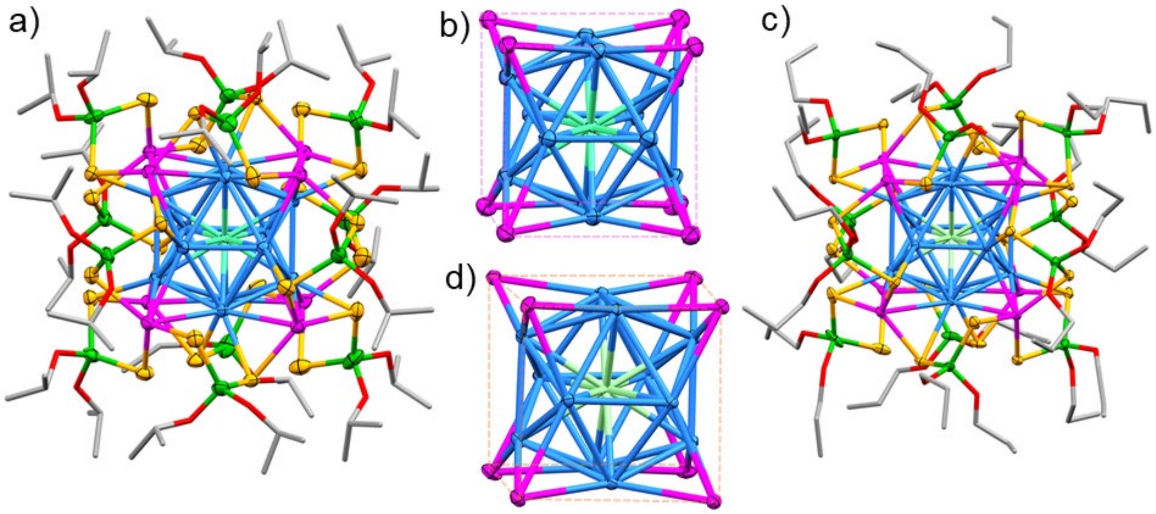

Supplementary Figure S11. (a) Total structure of [$PdAg_{20}\{Se_2P(O^iPr)_2\}_{12}$] (**3**) (isopropoxy groups are shown in capped sticks model), (b) Illustration of Pd@Ag_{20} metallic core in **3** with T_h symmetry, (c) Total structure of [$PdAg_{20}\{Se_2P(O^nPr)_2\}_{12}$] (**4**) (propoxy groups are shown in capped sticks model), (d) Illustration of Pd@Ag_{20} metallic core in **4** with T_h symmetry, (Colour code. Pd: light green; Ag_{ico}: blue, Ag_{cap}: pink; Se: orange; P: green).

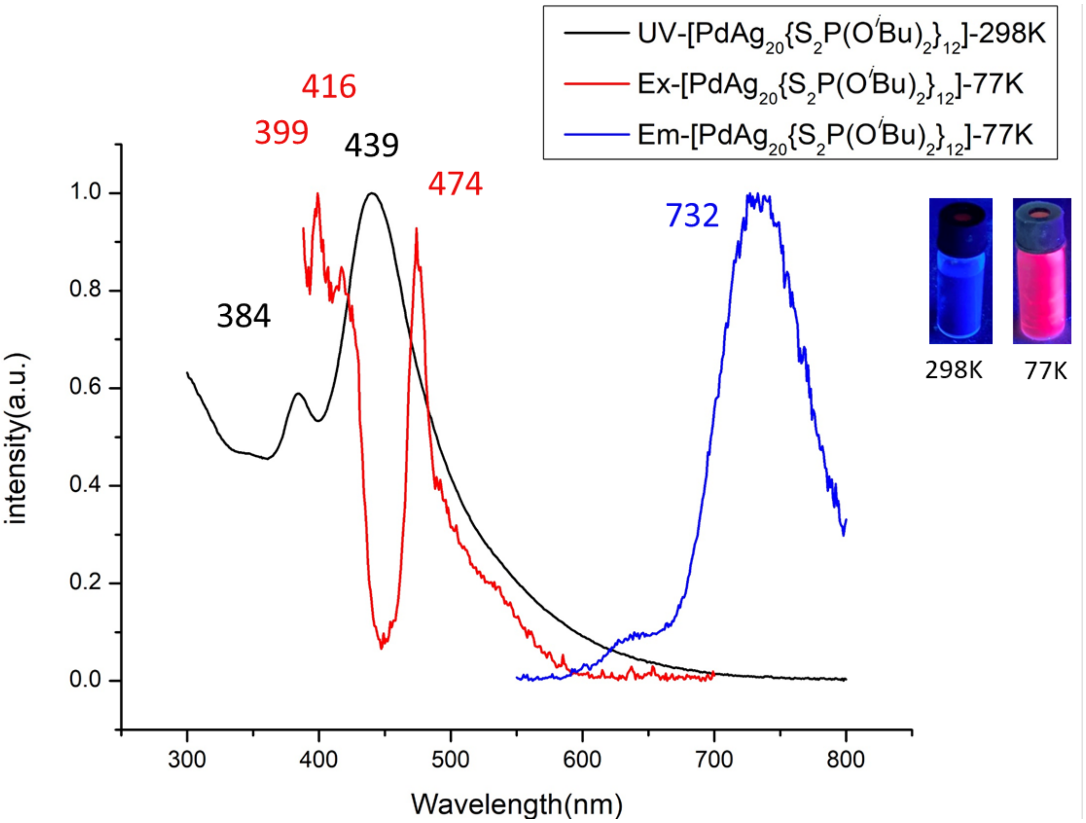

Supplementary Figure S12. UV-vis, excitation and emission spectra of compound **2b** in MeTHF.

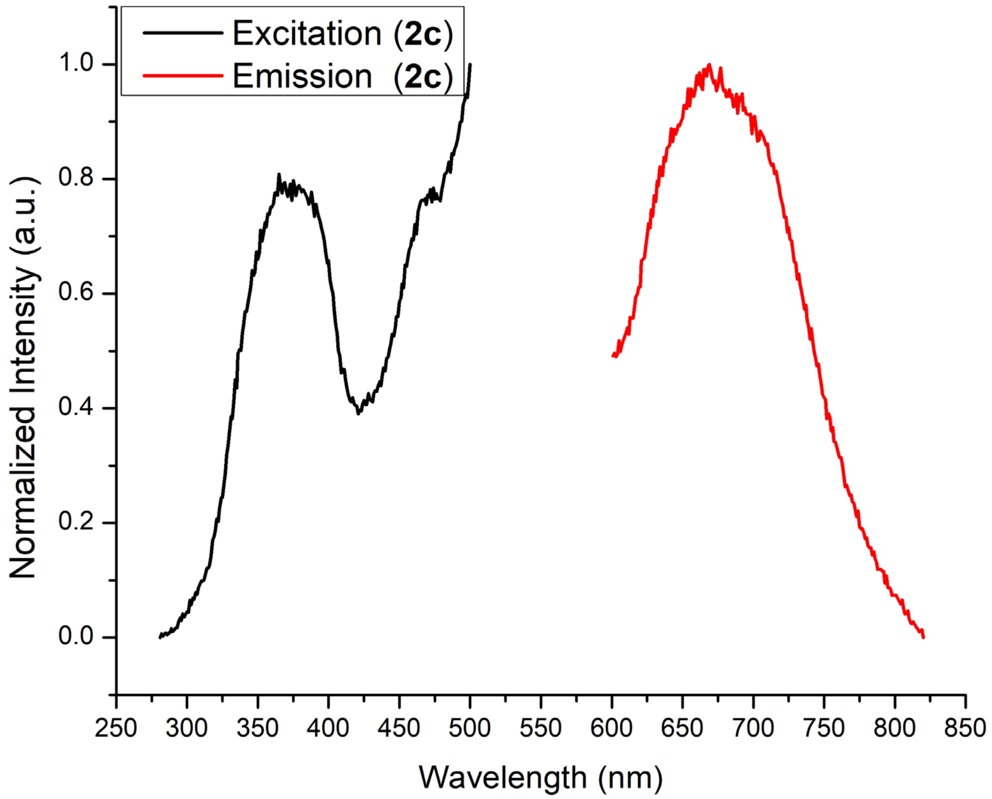

Supplementary Figure S13. Excitation (left) and emission (right) spectra of compound **2c** in MeTHF at 77K.

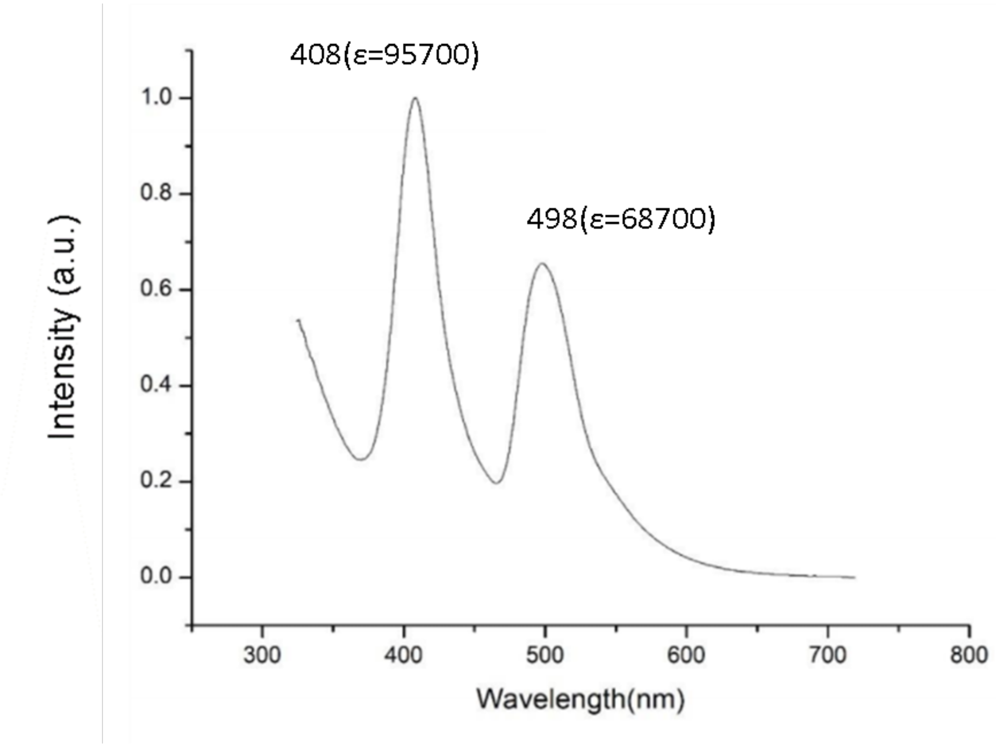

Supplementary Figure S14. UV–vis spectrum of **4** in 1 x 10^{-5} M CH_2Cl_2.

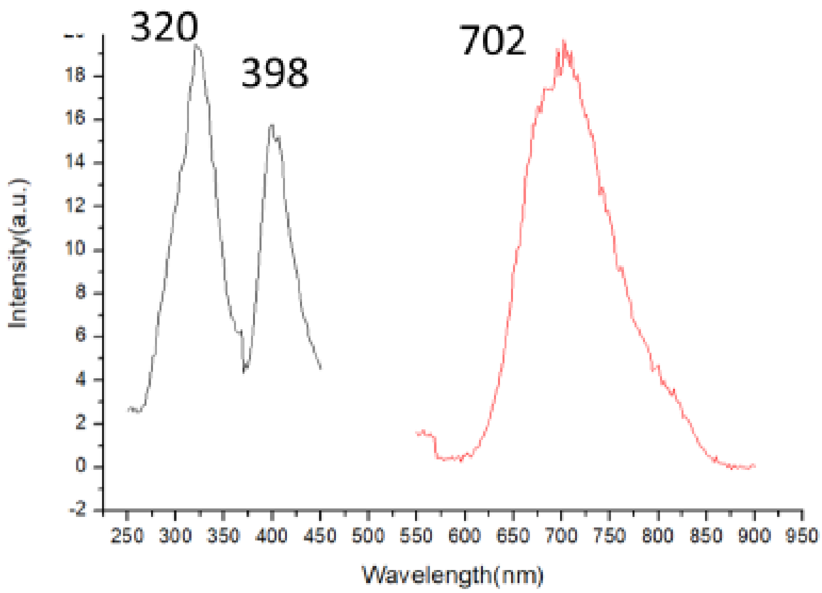

Supplementary Figure S15. Excitation and emission spectra of compounds **4** in MeTHF at 77K.

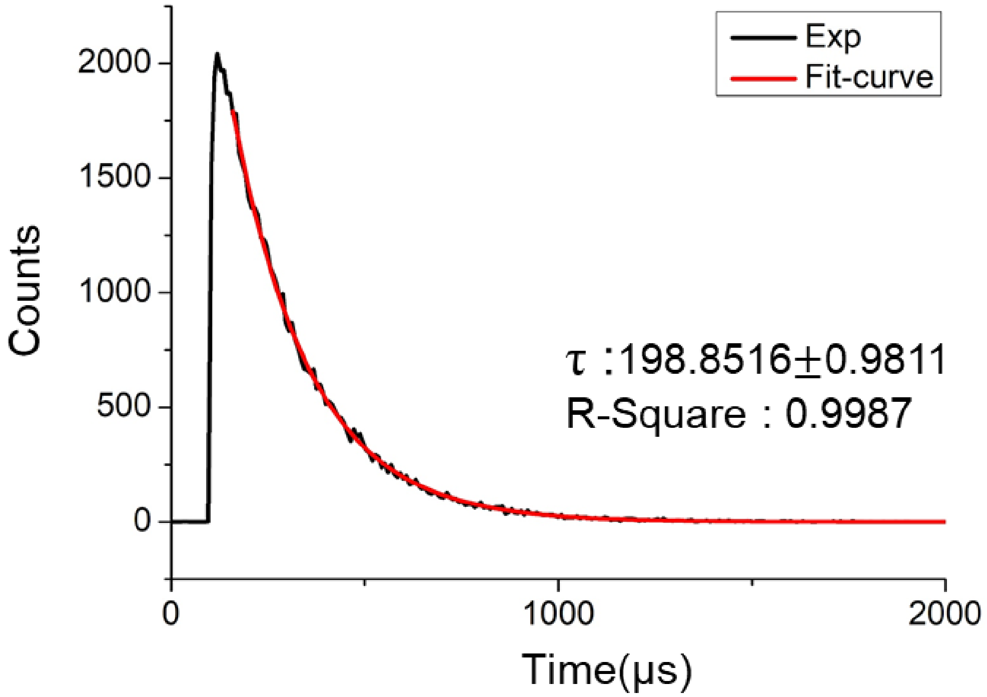

Supplementary Figure S16. Time-resolved photoluminescence spectrum of **2b** at 77K.

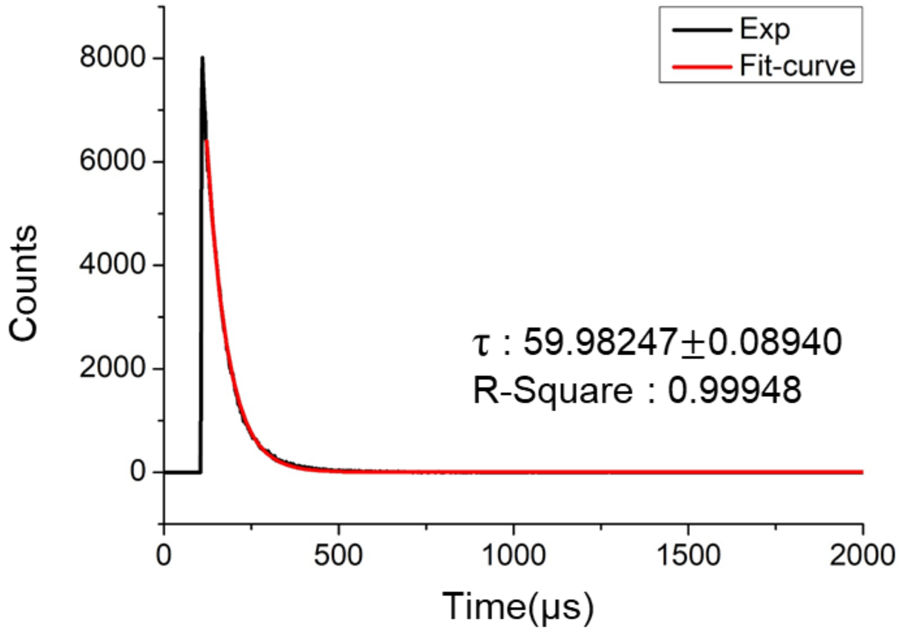

Supplementary Figure S17. Time-resolved photoluminescence spectrum of **2c** at 77K.

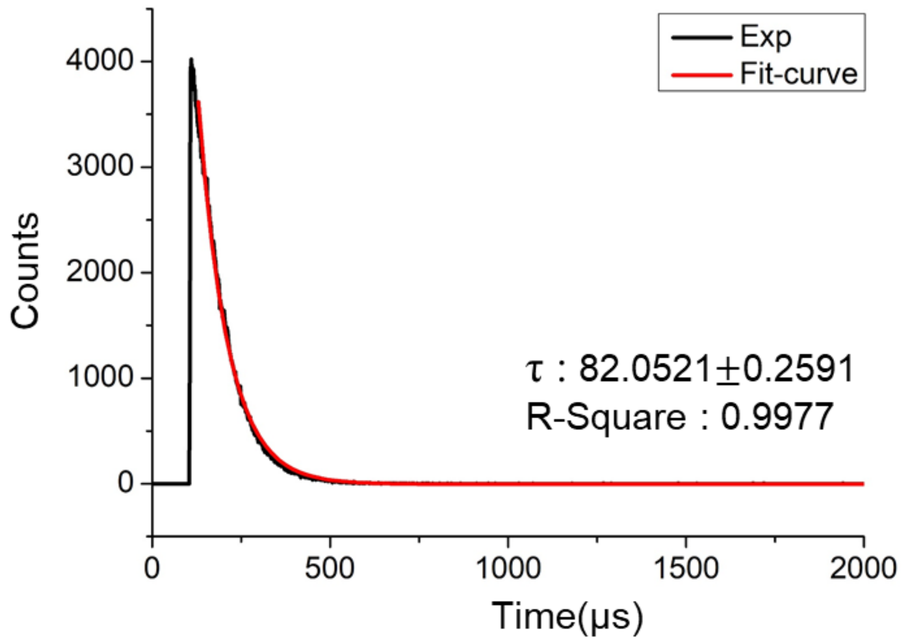

Supplementary Figure S18. Time-resolved photoluminescence spectrum of **4** at 77K.
