# Supplementary material for: Surface modifications of eight-electron palladium silver superatomic alloys
Source: Commun Chem. 2022 Nov 19;5:151. doi: 10.1038/s42004-022-00769-2 (PMC9814913; doi:10.1038/s42004-022-00769-2)
Supplement: Supplementary file 2 — Description of Additional Supplementary Files [file 42004_2022_769_MOESM2_ESM.pdf]

Description of Additional Supplementary Files

File name: Supplementary Data 1

Description: NMR spectra

File name: Supplementary Data 2

Description: Cartesian coordinates

File name: Supplementary Data 3

Description: X-ray Data
