# Supplementary material for: Surface modifications of eight-electron palladium silver superatomic alloys
Source: Commun Chem. 2022 Nov 19;5:151. doi: 10.1038/s42004-022-00769-2 (PMC9814913; doi:10.1038/s42004-022-00769-2)
Supplement: Supplementary file 3 — Supplementary data 1 [file 42004_2022_769_MOESM3_ESM.docx]

The NMR spectra of all palladium silver nanoclusters

**
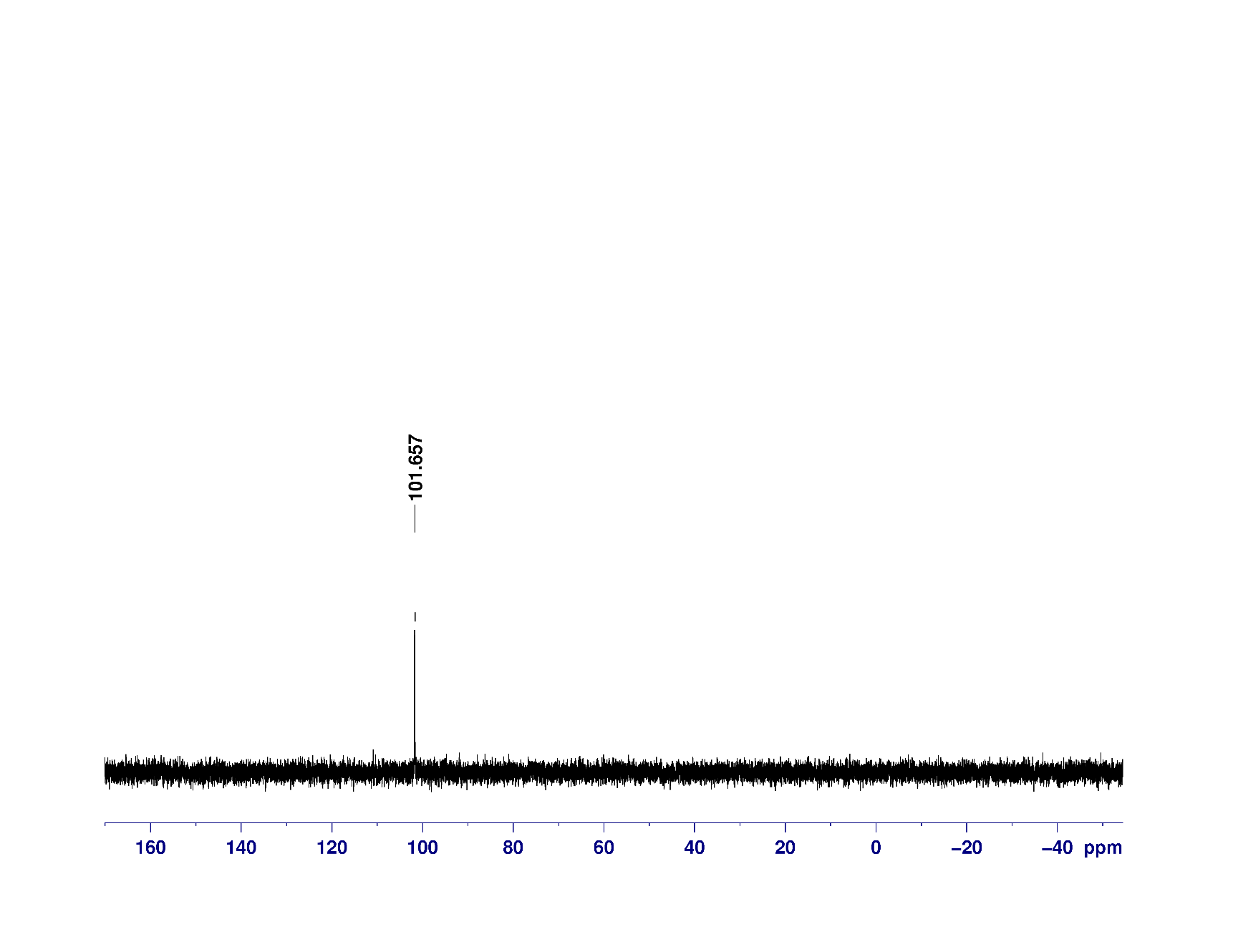
**

**Figure 1**. ^31^P{^1^H} (121.49 MHz, CDCl_3_) NMR spectrum of **2a**.


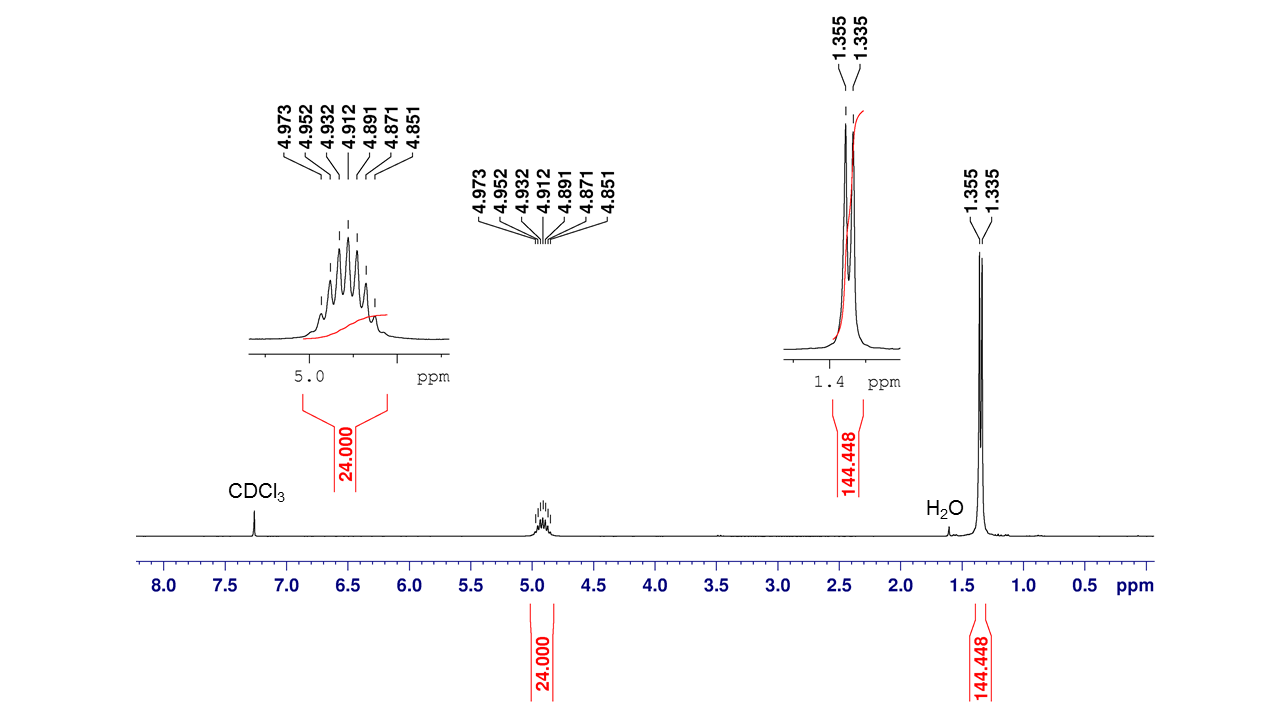


**Figure 2**. ^1^H NMR spectrum of **2a** (300 MHz, CDCl_3_).


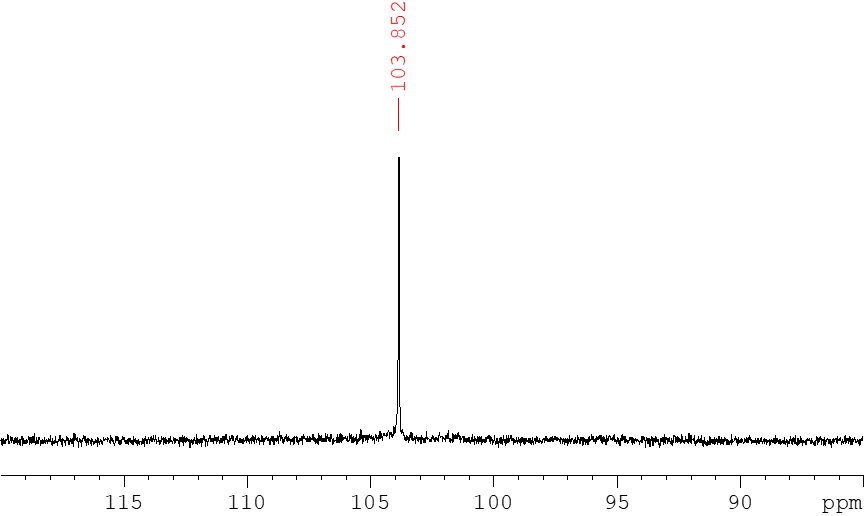


**Figure 3**. ^31^P{^1^H} (161.9 MHz, CDCl_3_) NMR spectrum of **2b**.


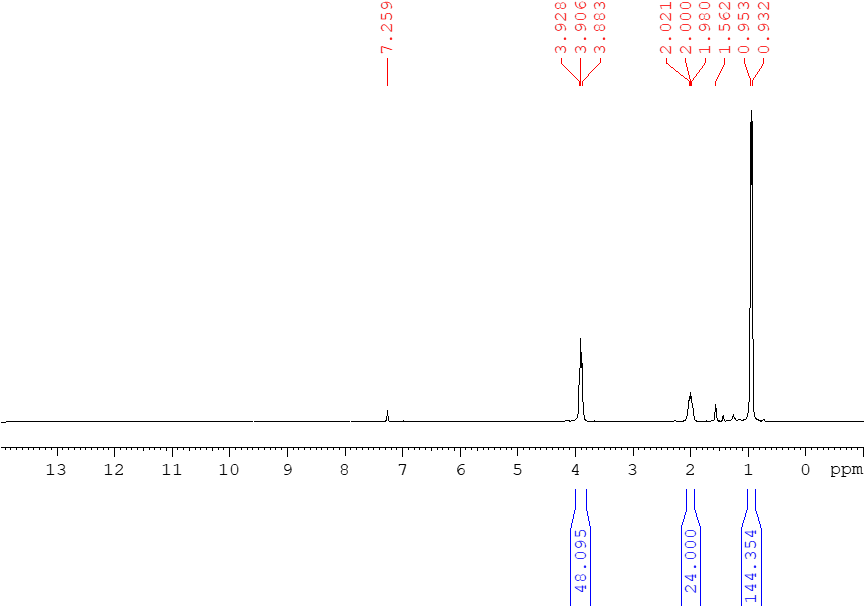


**Figure 4**. ^1^H NMR spectrum of **2b** (300 MHz, CDCl_3_).

**Figure 5**. ^31^P{^1^H} (121.49 MHz, CDCl_3_) NMR spectrum of **2c**.

**
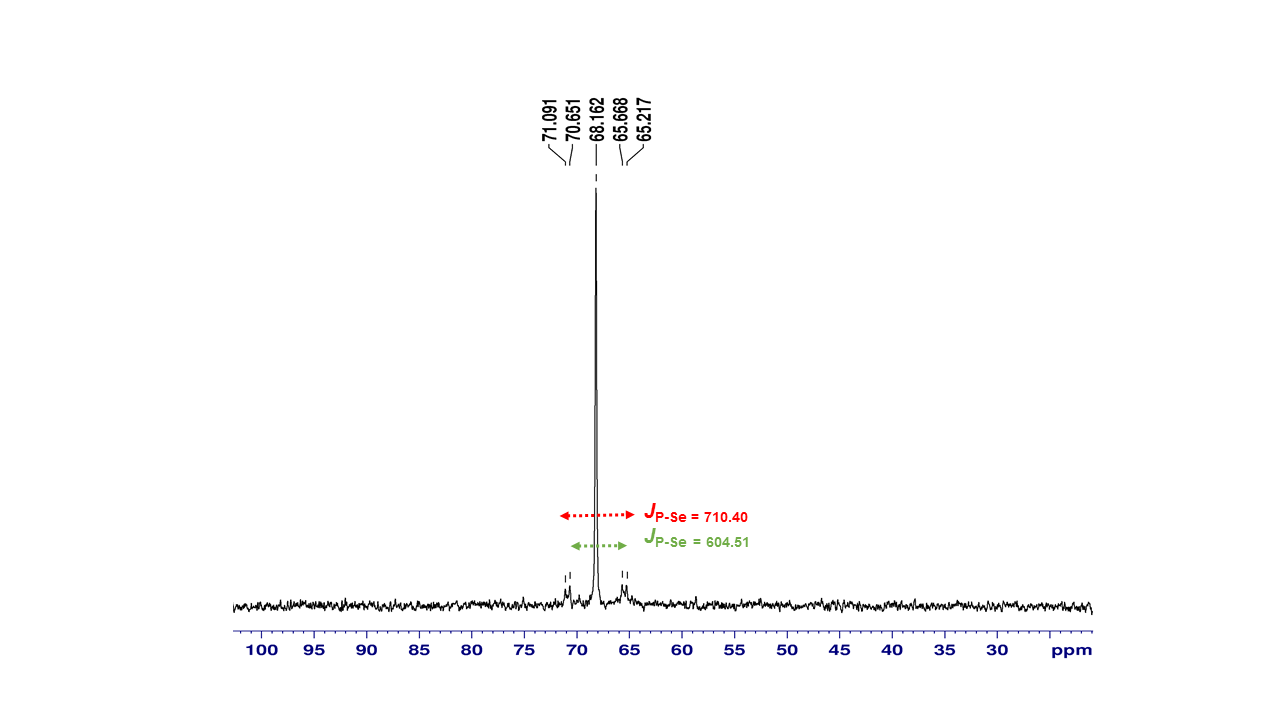
**

**Figure 6**. ^31^P{^1^H} NMR (161.9 MHz) spectrum of **3** in *d*_6_-acetone.


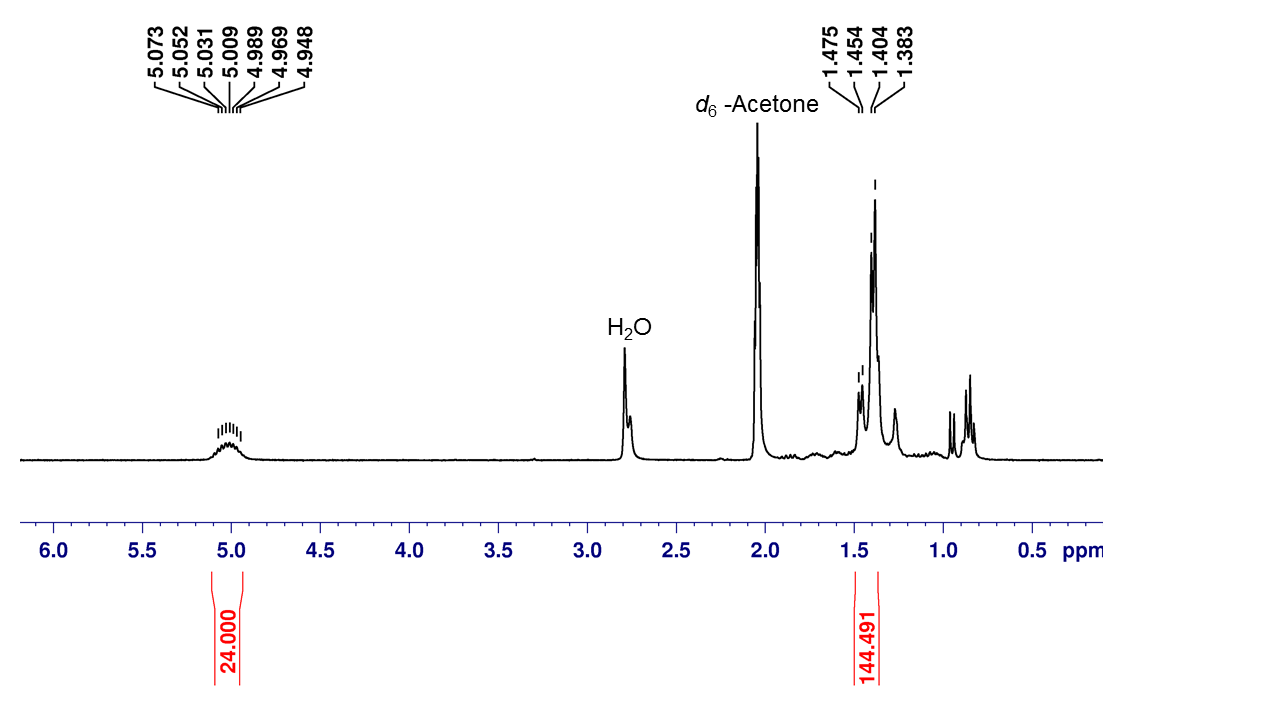


**Figure 7**. ^1^H NMR (400 MHz, *d*_6_-acetone) spectrum of **3**.

**Figure 8**. ^31^P{^1^H} NMR (161.9 MHz, *d*_6_-acetone) spectrum of **4**.

**Figure 9**. ^1^H NMR (400 MHz, CDCl_3_) spectrum of **4**.
